# Co-expression analysis identifies neuro-inflammation as a driver of sensory neuron aging in *Aplysia californica*

**DOI:** 10.1371/journal.pone.0252647

**Published:** 2021-06-11

**Authors:** N. S. Kron, L. A. Fieber

**Affiliations:** Department of Marine Biology and Ecology, Rosenstiel School of Marine and Atmospheric Science, University of Miami, Miami, FL, United States of America; Nanjing University, CHINA

## Abstract

Aging of the nervous system is typified by depressed metabolism, compromised proteostasis, and increased inflammation that results in cognitive impairment. Differential expression analysis is a popular technique for exploring the molecular underpinnings of neural aging, but technical drawbacks of the methodology often obscure larger expression patterns. Co-expression analysis offers a robust alternative that allows for identification of networks of genes and their putative central regulators. In an effort to expand upon previous work exploring neural aging in the marine model *Aplysia californica*, we used weighted gene correlation network analysis to identify co-expression networks in a targeted set of aging sensory neurons in these animals. We identified twelve modules, six of which were strongly positively or negatively associated with aging. Kyoto Encyclopedia of Genes analysis and investigation of central module transcripts identified signatures of metabolic impairment, increased reactive oxygen species, compromised proteostasis, disrupted signaling, and increased inflammation. Although modules with immune character were identified, there was no correlation between genes in Aplysia that increased in expression with aging and the orthologous genes in oyster displaying long-term increases in expression after a virus-like challenge. This suggests anti-viral response is not a driver of Aplysia sensory neuron aging.

## 1 Introduction

As an organ composed of long-lived cells, the brain is uniquely susceptible to the deleterious effects of aging, the outcome of which is often cognitive impairment [1,2]. Common hallmarks of brain aging include impaired metabolism, compromised proteostasis, mitochondrial dysfunction, and neuro-inflammation [3–5]. However, what causes these hallmark phenotypes is not well understood and still debated [6–8]. Due to the complexity of mammalian brains, invertebrate models are often employed for the study of aging in the nervous system at the neuronal level.

The marine model *Aplysia californica* is well suited for study of aging neurons. These mollusks live approximately one year in the wild and mariculture setting and have relatively simple nervous systems of only approximately 10,000 neurons grouped into well mapped circuits [9–11]. Studies on the life history and reflex behaviors of these animals in controlled environments have described weight loss and cognitive impairment in old age similar to that of other animals and humans [12–16]. Investigation of physiology and transcriptomics in easily identifiable neurons and neuron clusters during aging have elucidated neuronal correlates that underpin aging phenotypes at the behavior and organism level [17–23]. Indeed, it is the capacity for vertical integration of investigation, from molecular to behavioral, that makes Aplysia such an effective model for the aging nervous system. Previously, transcriptomic studies in aging sensory neurons of Aplysia identified signatures of common aging hallmarks, namely metabolic and proteostatic impairment [20,24]. However, due to the limitations of differential expression analysis (DEA), including log fold change thresholds and multiple comparison [25], the driving mechanism behind these transcriptional changes could not be identified. A common alternative analysis that can overcome these limitations is weighted gene correlation network analysis (*WGCNA*).

WGCNA is able to lessen the impact of correction for multiple tests by comparing changes in groups of genes, called modules, as opposed to individual genes and does not employ the strict fold change thresholds used in DEA [25,26]. This method can capture network level changes that integrate the small, coordinated changes of many genes that would otherwise be below the thresholds employed in DEA [27]. Furthermore, *WGCNA* allows for the identification of putative central driver genes in co-expression networks and inference of putative function for genes that are undescribed or have yet to be experimentally verified via guilt-by-association [26,28,29]. In this study we used *WGCNA* and eigengene network analysis to identify the central drivers of the transcriptional aging phenotype of Aplysia SN.

## 2 Methods

### 2.1 Experimental design

Sequencing data for this study were generated in our previous study [24]. RNA was extracted from *A*. *californica* Buccal S Cluster (BSC) and Pleural Ventral Caudal (PVC) sensory neurons across the adult aging spectrum (6–12 months).

### 2.2 Raw read processing

Raw, 150 base pair, paired end RNA reads from our previous study, available at the NCBI (PRJNA639857), were processed as described previously [24]. Briefly, raw reads were adapter trimmed and quality filtered using the *BBDUK* software [30] and then quantified by the *Salmon* software package [31] using the *Aplysia californica* reference transcriptome from the NCBI ftp site (AplCal3.0 GCF000002075.1).

*Salmon* derived transcript abundances were imported into the R statistical environment via the *tximport* R package [32]. Transcripts with a sum total abundance of less than 1 transcript per million (TPM) across all samples were considered not expressed and filtered out. The R package *geneFilter* was used to remove low variance transcripts using the function varFilter() with the default parameters var.func = IQR and var.cutoff = 0.5 to filter out transcripts with interquartile range (IQR) smaller than the median of all IQR in the expression data [33]. Surrogate variables were identified using the *SVA* R package [34]. Abundances were then variance stabilized using the vst() function from the *DESeq2* R package, with surrogate variables incorporated into the model design and the *blind* parameter set to *FALSE* to account of said surrogate variables *[35]*.

### 2.3 Co-expression analysis

Variance stabilized counts from BSC and PVC samples were used to construct sensory neuron type specific co-expression networks using the *WGCNA* R package. Briefly, Biweighted midcorrelation in *WGCNA* was used to construct adjacency matrices for each sensory neuron type independently. A soft power of 16 was used for both PVC and BSC networks to achieve at least 0.8 metric for scale free topology and to minimize mean connectivity (S1 Fig). Adjacency matrices were used to construct signed topological overlap matrices (TOM) for each sensory neuron type. The sensory neuron type-specific TOMs were centered and scaled and combined to make a consensus TOM by taking the minimum of the two modules. Transcripts were hierarchically clustered with the average method using a distance metric of 1-consensus TOM and a minimum module size of 30.

Initial module eigengenes were hierarchically clustered to determine module similarity. Modules with a branch height of 0.25 or less were deemed insufficiently different from their neighbors and merged. Module eigengenes for merged modules were then recalculated (S2 and S3 Figs). Consensus module eigengenes were then correlated to animal chronological age.

Individual transcripts in each module were then correlated to the module eigengene, referred to as module membership, as a measure of the transcript centrality.

Similarly, transcripts were correlated with chronological age, referred to as transcript-age significance (TAS), as a measure of the influence of chronological age on the expression of that transcript. Correlation of module membership with TAS of all transcripts within a module (MM-TAS) was calculated to describe the influence of chronological age on the module as a whole. Only modules with high magnitude (|Pearson cor| ≥ 0.5) and high degree of significance (p ≤ 0.01) of MM-TAS were considered for further downstream analysis. Module hub transcripts were identified using the *WGCNA* function chooseTopHubInEachModule().

### 2.4 Module enrichment analysis

Transcript sets of each module were tested for enrichment for Kyoto Encyclopedia of Genes and Genomes (KEGG) canonical pathways using the *clusterProfiler* R package [36].

All software used and version information is available in Supplementary Table S1 Table and Supplementary Information **S1 File**. All scripts used for this analysis can be found in the following GitHub repository: [https://github.com/Nicholas-Kron/Kron_Cohort77_CoExpression_Analysis]

### 2.5 Comparison to immune response in *Crassostrea gigas*

To assess whether the observed module immune signatures suggested mapping to KEGG orthology and the UNIPROT human proteome represented a bona fide molluscan immune signature, module transcript sets were compared to differential expression resulting from immune challenge in the pacific oyster *Crassostrea gigas* by Lafont et al (2020) [37].

The Aplysia RefSeq proteome for the current genome build (AplCal3.0, GCF_000002075.1) was downloaded from the RefSeq database. Similarly, the proteome for the *C*.*gigas* genome build used by Lafont et al (2020, oyster_v9, GCA_000297859.1) [37] was downloaded from the Ensemble FTP site. The Aplysia proteome was BLASTed against local BLAST database built from said *C*.*gigas* proteome using an e value cutoff of less than 0.001 and selecting only the top hit.

The resultant putative protein orthologs between Aplysia and *C*.*gigas* were then mapped to their respective gene and transcripts identifiers using the respective genome build gene feature format annotation files (AplCal3.0_genomic.gff version 1.21 and oyster_v9.49.gff3). The new Aplysia to *C*. *gigas* mapping file was used to determine the proportion of each co-expression module that mapped to genes differentially expressed in response to immune challenge either due to exposure to poly(I·C) priming, viral challenge, or both [37]. Enrichment for *C*. *gigas* orthologs in each module was calculated by Fisher’s exact test with Bonferroni multiple test correction in R using the *dhyper* function.

## 3 Results

### 3.1 Filtering

Removal of transcripts with zero total count and variance filtering with the *geneFilter* package yielded 11,703 analysis ready transcripts, out of approximately 12,000 transcripts expressed in these sensory neuron types described previously [24]. SVA identified one surrogate variable which was included in the DESeq2 model design.

### 3.2 Clustering

**Fig 1:**
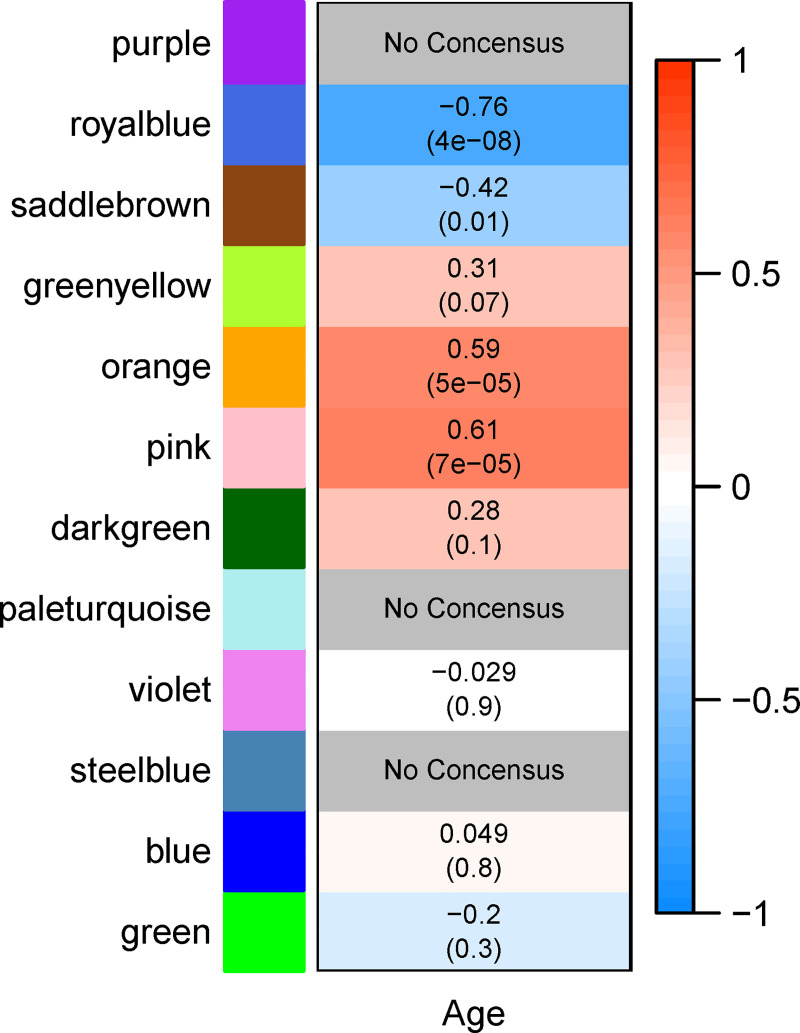
Correlation in *Aplysia californica* sensory neurons between consensus co-expression modules and animal age. Each module is arbitrarily assigned a color to assist in reference. This color is denoted on the heatmap row labels of Fig 1 and referred to throughout the figures and tables. Each cell of the heatmap represents the Pearson correlation between a module eigengene (row) and age (column). The upper value within a cell represents the magnitude of correlation. The lower value in parentheses in each cell represents the p-value of the correlation. Cell color denotes direction of correlation (red = positive, blue = negative) and saturation represents magnitude of correlation, with greater magnitude of correlation (top value in each cell) represented by higher saturation. Modules for which sign of eigengene correlation with age between PVC and BSC was inconsistent were colored grey and marked as “No Consensus” due to lack of consensus. *Orange* and *pink* modules are significantly correlated with age, while *royalblue* and *saddlebrown* are significantly anti-correlated.

Hierarchical clustering of transcripts and module merging resulted in 12 consensus co-expression modules, which were assigned arbitrary color names by *WGCNA*. A thirteenth module wastebasket module was assigned the “grey” designation and not evaluated in downstream analysis. The *royalblue*, *saddlebrown*, *orange*, and *pink* module eigengenes exhibited significant correlation with chronological age (p ≤ 0.05; Fig 1). Module-trait correlations in both PVC and BSC individually can be found in Supplementary Figure **S4 Fig**. The hub transcript for each module can be found in Table 1.

**Table 1:** Co-expression modules identified in *Aplysia californica* sensory neurons.

Module	Module n	Hub gene RefSeq ID	Human Ortholog	Ortholog Name
**blue**	1581	XM_013088909.1	MGA	MAX gene-associated protein
**darkgreen**	225	XM_013082889.1	RPA2	Replication protein A 32 kDa subunit
**green**	2387	NM_001204703.1	GNAO1	G-protein G(o) subunit alpha
**greenyellow**	329	XM_005110768.2	ZNFX1	NFX1-type zinc finger-containing protein 1
**orange**	166	XM_005096841.2	CREB3L3	Cyclic AMP-responsive element-binding protein 3-like protein 3
**paleturquoise**	41	XM_013087385.1	-	-
**pink**	1255	XM_005111489.2	NFKBIA	NF-kappa-B inhibitor alpha
**purple**	3036	XM_005095177.2	PSMB7	Proteasome subunit beta type-7
**royalblue**	561	XM_005101095.2	-	-
**saddlebrown**	65	XM_005089315.2	-	-
**steelblue**	64	XM_013083399.1	EBF3	Transcription factor COE3
**violet**	36	XM_013085790.1	ATXN10	Ataxin-10

Modules were identified using the weighted gene correlation network analysis (*WGCNA*) R package. The most connected transcript, called the hub gene, is listed by its RefSeq identifier, as well as its BLASTx assigned human ortholog. Hub transcripts with a “-” in the Human Ortholog and Ortholog Name columns could not be mapped to any known human protein, and thus are of unknown function.

### 3.3 Modules of interest

The expression of each transcript in a module was correlated with the module eigengene, called module membership (MM), and with age, called transcript-age significance (TAS). Modules for which MM and TAS were highly correlated (|Pearson cor| ≥ 0.5, p ≤ 0.01) were investigated further.

**Fig 2:**
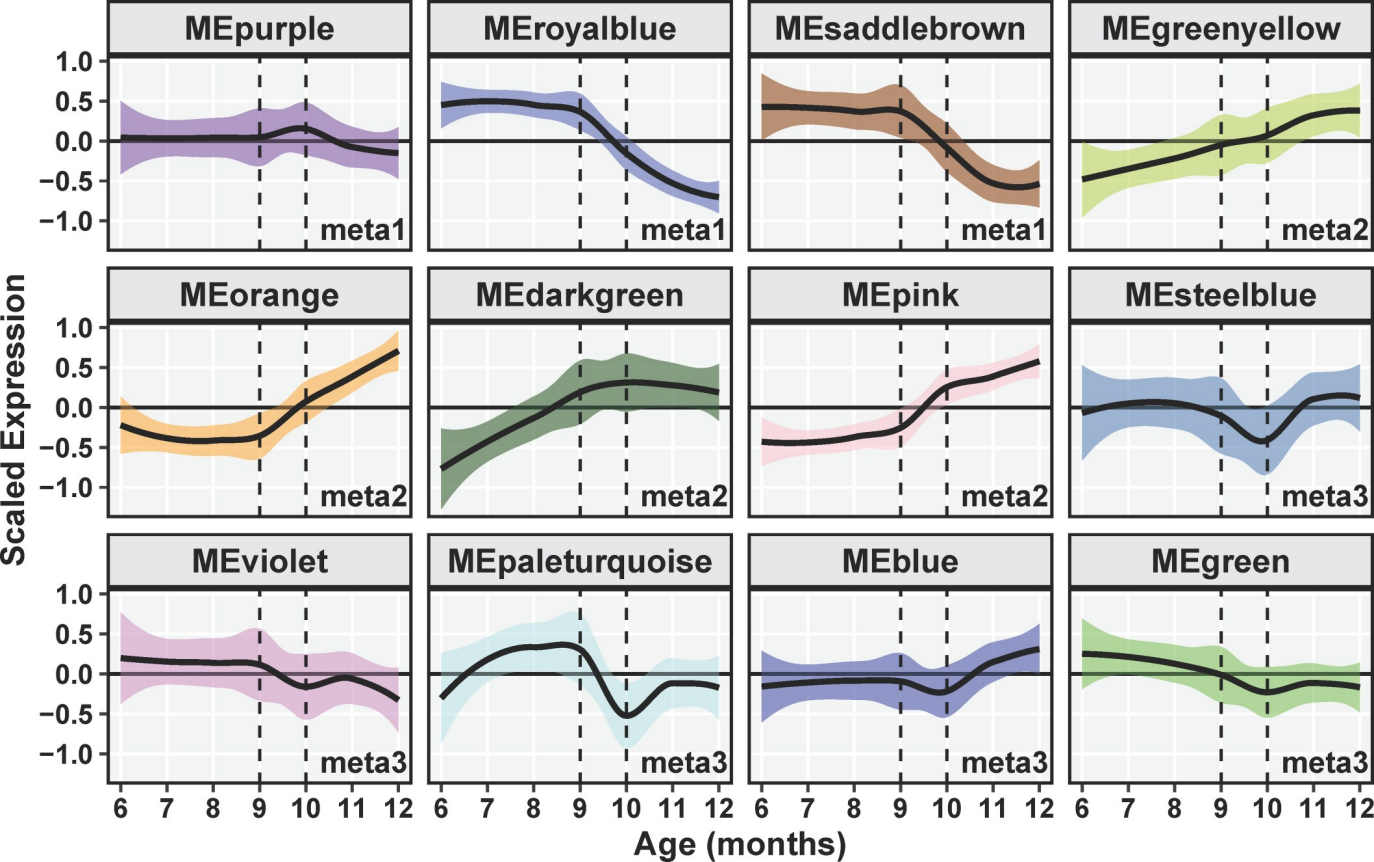
Expression trajectories of consensus co-expression module eigengenes of *Aplysia californica* sensory neurons over the adult lifespan. Each cell represents the mean centered and variance scaled expression of a module eigengene, with the solid line the monthly average with colored bounding area representing the standard error. Dotted lines highlight the transition at age 9–10 months, during which most module eigengenes exhibit perturbations of their expression trends.

**Fig 3:**
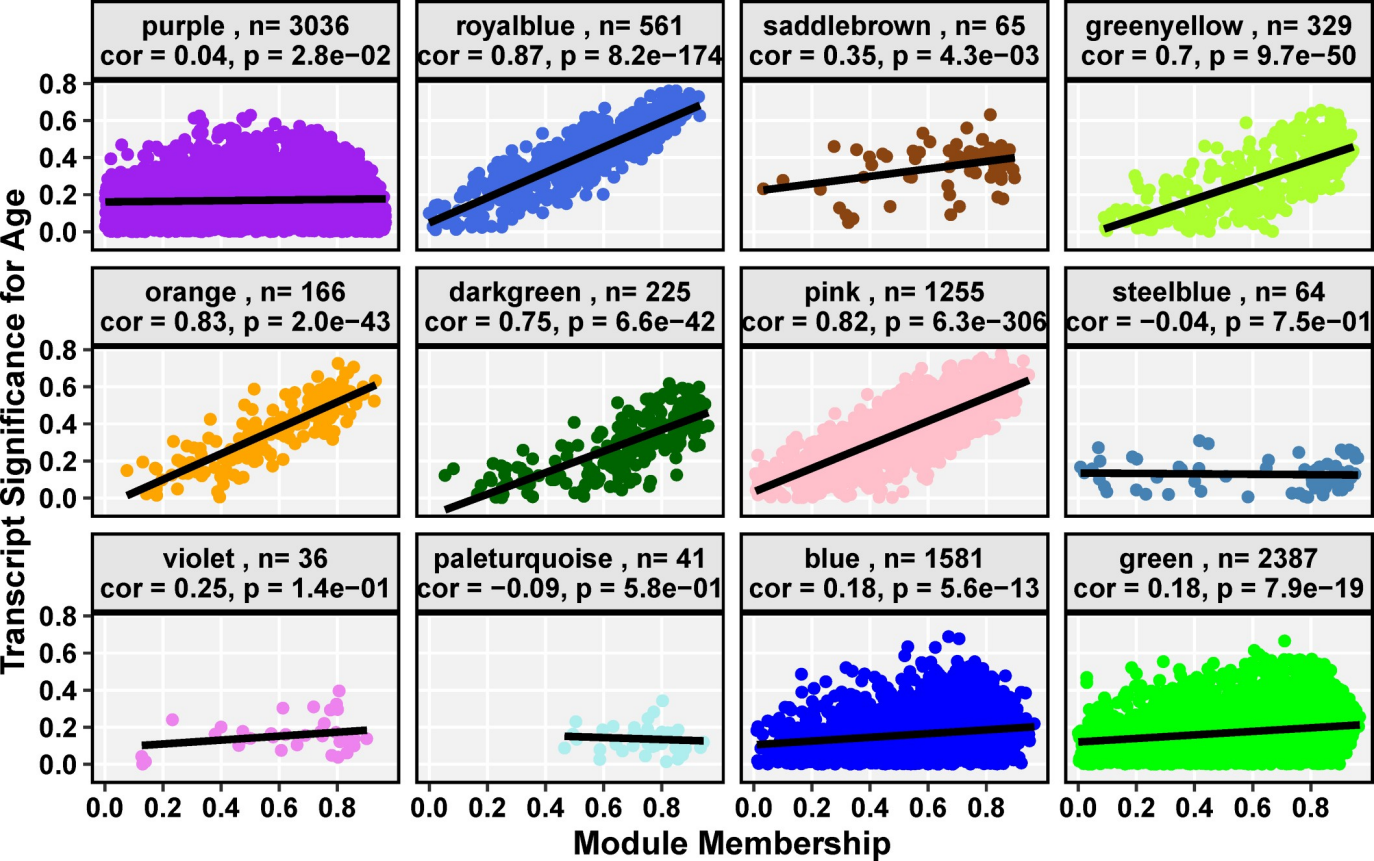
Trait significance–module membership correlation (TS-MM) for each co-expression module of *Aplysia californica* sensory neurons over the adult lifespan. The x-axis of each cell is the Pearson correlation of the expression of a transcript and the module eigengene. The y-axis of each cell is the Pearson correlation of the expression of a transcript and chronological age in months. Each module transcript is plotted as a colored point, while the line of best fit, which represents the TS-MM, is rendered in black. Header strips detail the module name, the number of transcripts in that module (n), the TS-MM Pearson correlation value, and the p-value significance of that correlation for each module as calculated by the WGCNA R package. The *darkgreen*, *greenyellow*, *orange*, *pink*, and *royalblue* modules have a significant TS-MM ≥ 0.7.

Both the *royalblue* and *saddlebrown* modules were significantly anti-correlated with age. Eigengene expression trend of both modules was stable until age nine months when expression decreased monotonically (Fig 2). Module membership was highly correlated with the transcript-age significance for age (TAS) in the *royalblue* module (Pearson cor ≥ 0.8, p ≤ 0.001, Fig 3). However, MM-TAS was not strongly correlated for the *saddlebrown* module and thus this module was not investigated further (Pearson cor = 0.35, p = 0.004, Fig 3).

Eigengenes of the *pink*, *orange*, *darkgreen*, and *greenyellow* modules exhibited increasing expression trends with increasing chronological age for at least some portion of the age span (Fig 2). The *pink* and *orange* module eigengenes were significantly correlated with chronological age (p ≤ 0.05, Fig 1) and both modules exhibited high MM-TAS correlation (Pearson cor ≥ 0.7, p ≤ 0.001; Fig 2). Notably, the expression profile of the *orange* and *pink* eigengenes resemble the *royalblue* and *saddlebrown* eigengene mirrored over the x-axis, exhibiting a linear increase in expression after age 9 months. The *greenyellow* module exhibited a linear increase in eigengene expression with age across the entire aging span, while *darkgreen* exhibited increasing expression from ages 6–9 months (Fig 2). While the module eigengenes of these two modules were not significantly correlated with age, both modules exhibited strong MM-TAS correlation (Pearson cor ≥ 0.7, p ≤ 0.001; Fig 3). This suggests aging strongly affects the central regulators of these modules, a notion sufficiently interesting to justify further investigation of these modules.

Many of the age associated modules overlapped with transcriptional trajectories of transcripts differentially expressed in aging in our previous study (Supplementary table S2 Table).

### 3.4 Enrichment analysis of modules of interest

#### 3.4.1 Royalblue

**Fig 4:**
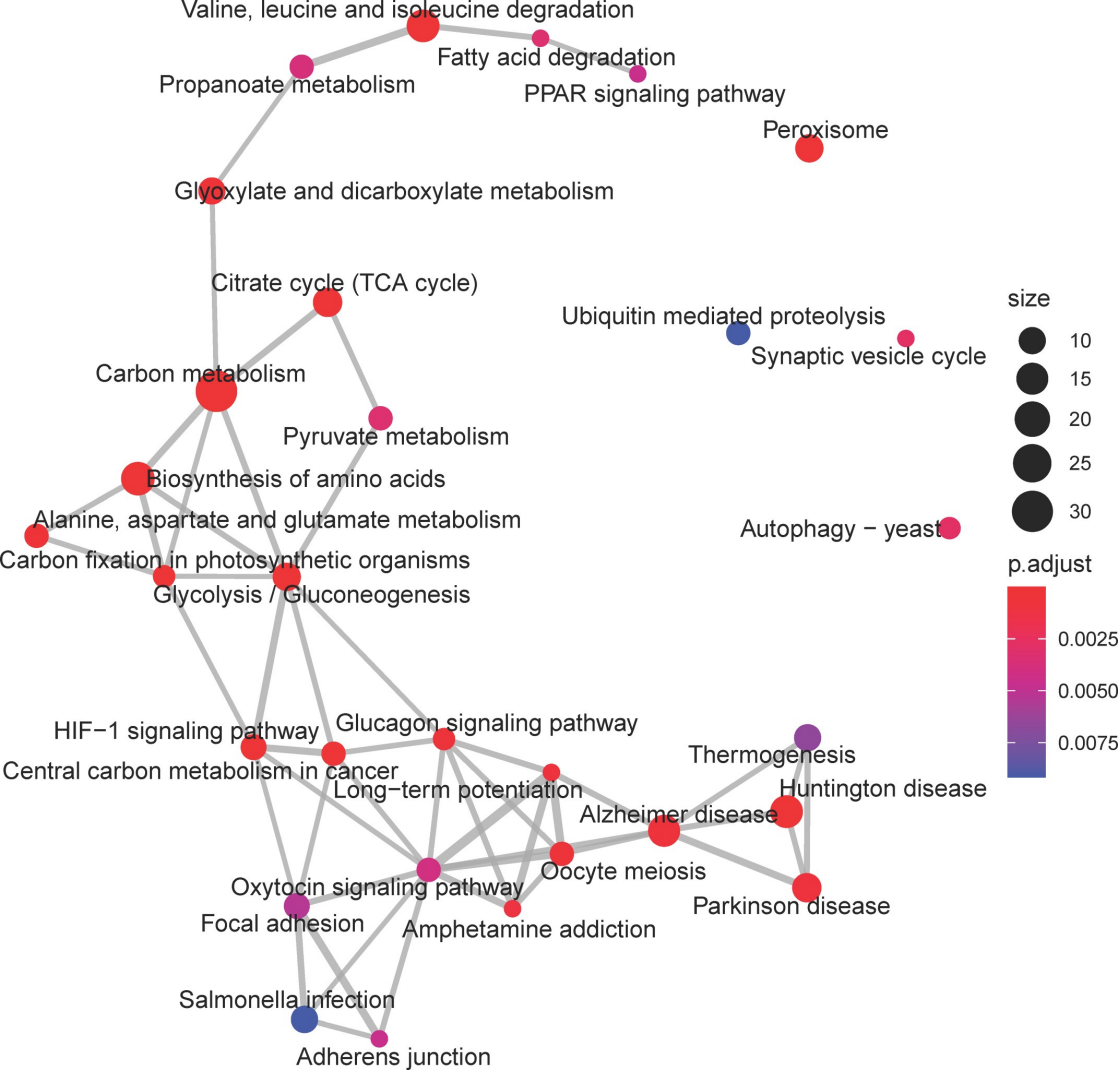
Enrichment map of Kyoto Encyclopedia of Genes and Genomes (KEGG) pathways for the *royalblue* consensus co-expression module from *Aplysia californica* sensory neurons. Each node represents a KEGG pathway, with node size representing the number of transcripts annotated to that pathway, and color denoting the significance of that enrichment (brighter red is most significant). KEGG pathways with overlapping transcripts sets are connected by grey lines, or edges. Edge width is determined by the number of overlapping transcripts. This module exhibits a high degree of gene set overlap between most of the enriched KEGG pathways. Metabolic pathways such as *TCA cycle*, *Glycolysis*, and *fatty acid degradation* are among the largest and most significantly enriched. The module eigengene trend of this module was negatively correlated with age, indicating downregulation of these pathways.

The *royalblue* module, which exhibited decreasing eigengene expression trend with age, was enriched for a diverse set of KEGG pathways (Fig 4). Most prominent were the canonical energy metabolism pathways of *glycolysis* (ko00010), *TCA cycle* (ko00020), and *fatty acid metabolism* (ko01212). Amino acid metabolism related pathways such as *biosynthesis of amino acids* (ko01230) and *alanine*, *aspartate*, *and glutamate metabolism* (ko00250 were also enriched. Another set of enriched KEGG pathways represent elements important to neuronal function such as *synaptic vesicle cycle* (ko04721), *long-term potentiation* (ko04720), *MAPK signaling* (ko04013), and *calcium signaling* (ko04020). Several enriched pathways represent human neurodegenerative diseases that sit at the nexus of neuronal dysfunction and metabolic failure such as *Alzheimer’s disease* (ko05010), *Huntington’s disease* (ko05016), and *Parkinson’s disease* (ko05012).

Orthologs also involved in anterograde or retrograde movement of cellular cargo featured prominently among the transcripts with highest module membership in this module. Metabolic enzymes associated with TCA cycle, glycolysis, and mitochondrial fatty acid beta oxidation were also prominent. Reactive oxygen species (ROS) detoxification enzymes and other mitochondrial homeostasis maintenance orthologs were also present (Table 2).

**Table 2:** Selection of transcripts with highest correlation to transcript co-expression module eigengene (module membership, MM) in the *royalblue* consensus module identified in *Aplysia californica* sensor neurons by *WGCNA*.

Refseq ID	MM	MM p value	TAS	TAS p value	Human Ortholog	Ortholog Name	Ortholog Function
*XM_005096347*.2**	0.93	9.9E-33	-0.63	2.95E-09	DCTN6	Dynactin subunit 6	Transport of cellular cargo [38]
*XM_005105237*.1**	0.93	6.4E-32	-0.73	2.46E-13	GPI	Glucose-6-phosphate isomerase	glycolysis, neurotrophic factor [39]
*XM_005093202*.2**	0.89	2.8E-25	-0.70	7.42E-12	NAPG	Gamma-soluble NSF attachment protein	Required for vesicular transport between the endoplasmic reticulum and the Golgi apparatus. (UniProt)
*XM_005101715*.2**	0.88	1.1E-24	-0.70	6.98E-12	EXOC2	Exocyst complex component 2	Component of exocyst complex (UniProt)
*XM_005096859*.2**	0.86	1.1E-22	-0.67	1.09E-10	GPX4	Phospholipid hydroperoxide glutathione peroxidase	Antioxidant [40]
*XM_005089329*.2**	0.86	3.2E-22	-0.71	1.84E-12	MAP2K1	Dual specificity mitogen-activated protein kinase kinase 1	MEK 1
*XM_005105342*.2*	0.85	2.2E-21	-0.76	5.45E-15	SNX30	Sorting nexin-30	Possibly intracellular trafficking (UniProt)
*XM_005103941*.2*	0.84	5.9E-21	-0.55	4.21E-07	SDHAF2	Succinate dehydrogenase assembly factor 2, mitochondrial	Assembly and function of succinate dehydrogenase complex [41]
*XM_013079678*.1*	0.84	1.0E-20	-0.66	1.71E-10	VAPA	Vesicle-associated membrane protein-associated protein A	vesicular transport between the endoplasmic reticulum and the Golgi apparatus [42]
*XM_005106229*.2**	0.84	2.9E-20	-0.57	1.83E-07	ACADS	Short-chain specific acyl-CoA dehydrogenase, mitochondrial	catalyze the first step of mitochondrial fatty acid beta-oxidation [43]
*XM_005112738*.2**	0.83	5.4E-20	-0.59	4.64E-08	ENO1	Alpha-enolase	glycolysis
*XM_013088411*.1**	0.83	6.5E-20	-0.59	3.18E-08	CCDC151	Coiled-coil domain-containing protein 151	dynein arm assembly [44]
*XM_005098999*.2**	0.83	8.4E-20	-0.61	7.88E-09	PARK7	Protein/nucleic acid deglycase DJ-1	Antioxidant, neuroprotection [45–47]
*XM_005096727*.2**	0.83	2.7E-19	-0.61	1.13E-08	GDAP1	Ganglioside-induced differentiation-associated protein 1	Regulator of mitochondrial network [48]
*XM_005111161*.*2*	0.79	7.9E-17	-0.56	2.47E-07			
*XM_005109966*.2**	0.82	3.2E-19	-0.76	6.07E-15	HADHA	Trifunctional enzyme subunit alpha, mitochondrial	catalyzes the last three of the four reactions of the mitochondrial beta-oxidation pathway [49]
*XM_005098946*.*2*	0.82	1.3E-18	-0.54	7.55E-07	C1orf194	Uncharacterized protein C1orf194	Ca^2+^ homeostasis [50]
*XM_013081239*.1**	0.82	1.3E-18	-0.53	1.23E-06	PGK1	Phosphoglycerate kinase 1	glycolysis
*XM_005089581*.2*	0.82	1.5E-18	-0.56	2.27E-07	EFCAB1	EF-hand calcium-binding domain-containing protein 1	Also called calaxin, binds Ca^2+^ and dynein [51]
*XM_005092435*.*2*	0.81	1.9E-18	-0.57	1.84E-07	CHMP6	Charged multivesicular body protein 6	ESCR-III complex, endosomal cargo sorting [52]
*NM_001204580*.1*	0.81	2.1E-18	-0.53	1.59E-06	CALM2	Calmodulin-2 (Fragment)	Ca^2+^ homeostasis [53]
*XM_005104646*.2**	0.81	2.2E-18	-0.55	4.29E-07	SOD2	Superoxide dismutase [Mn], mitochondrial	ROS defense [54]
*XM_005090003*.*2*	0.81	3.1E-18	-0.57	1.39E-07	CAT	Catalase	ROS defense [55]
*XM_005097336*.2*	0.81	3.2E-18	-0.55	3.87E-07	BLOC1S1	Biogenesis of lysosome-related organelles complex 1 subunit 1	anterograde transport [56]
*XM_005089746*.2**	0.81	3.8E-18	-0.58	5.48E-08	PGAM2	Phosphoglycerate mutase 2	glycolysis
*XM_005108968*.2**	0.81	4.0E-18	-0.64	1.09E-09	GPS2	G protein pathway suppressor 2 (Fragment)	mitochondrial retrograde signaling, mitochondrial biogenesis, transcriptional activator of nuclear-encoded mitochondrial genes [57]
*NM_001280826*.1**	0.81	5.0E-18	-0.57	1.90E-07	GAPDH	Glyceraldehyde-3-phosphate dehydrogenase	glycolysis
*XM_005097828*.2**	0.81	5.5E-18	-0.57	1.92E-07	PDHB	Pyruvate dehydrogenase E1 component subunit beta, mitochondrial	pyruvate dehydrogenase
*XM_005104734*.2**	0.81	7.3E-18	-0.55	3.70E-07	SDHA	Succinate dehydrogenase [ubiquinone] flavoprotein subunit, mitochondrial	TCA and OXPHOS
*XM_005100966*.2**	0.80	1.9E-17	-0.62	6.83E-09	DECR2	Peroxisomal 2,4-dienoyl-CoA reductase	mitochondrial fatty acid beta-oxidation [58]
*XM_013090573*.1*	0.80	2.1E-17	-0.56	2.64E-07	DLST	Dihydrolipoyllysine-residue succinyltransferase component of 2-oxoglutarate dehydrogenase complex, mitochondrial	TCA
*XM_005091339*.2**	0.80	2.2E-17	-0.61	8.96E-09	ETFA	Electron transfer flavoprotein subunit alpha, mitochondrial	Electron acceptor, mitochondrial fatty acid beta-oxidation [59]
*XM_013079116*.1*	0.80	2.3E-17	-0.54	6.66E-07	SYT4	Synaptotagmin-4	Retrograde signaling, endocytosis, Ca^2+^ sensing [60,61]
*XM_005106740*.2**	0.80	2.8E-17	-0.64	8.87E-10	FUNDC1	FUN14 domain-containing protein 1	mitochondrial maintenance [62]
*XM_005112721*.2**	0.80	3.6E-17	-0.59	5.11E-08	KCNC2	Potassium voltage-gated channel subfamily C member 2	ion channel [63]
*XM_013088705*.*1*	0.80	4.2E-17	-0.52	2.53E-06	MFN2	Mitofusin-2	mitochondrial fusion [64,65]
*XM_005105274*.*2*	0.79	4.8E-17	-0.63	3.04E-09	CCDC39	Coiled-coil domain-containing protein 39	Inner dynein arm assembly [66]
*XM_013084603*.1*	0.79	6.4E-17	-0.62	6.02E-09	VPS26B	Vacuolar protein sorting-associated protein 26B	Endosome retromer complex [67,68]
*XM_013079609*.1**	0.79	7.4E-17	-0.56	3.10E-07	IDH3G	Isocitrate dehydrogenase [NAD] subunit gamma, mitochondrial	TCA
*XM_005091388*.2**	0.79	8.9E-17	-0.68	5.00E-11	NXNL2	Nucleoredoxin-like protein 2	ROS defense, neurotrophic factor [69]
*XM_013087736*.1*	0.79	1.0E-16	-0.52	2.89E-06	NDUFAF2	NADH dehydrogenase [ubiquinone] 1 alpha subcomplex assembly factor 2	Mitochondrial complex I assembly and function [70,71]
*XM_005090280*.2**	0.79	1.6E-16	-0.68	2.75E-11	PDCD6	Programmed cell death protein 6	calcium sensor, ER to golgi transport, interacts with ESCRT-III [72]
*XM_005097549*.2*	0.78	2.5E-16	-0.45	5.60E-05	FMC1	Protein FMC1 homolog	Plays a role in the assembly/stability of the mitochondrial membrane ATP synthase [73]
*XM_013084592*.1**	0.78	8.4E-16	-0.56	2.79E-07	ACO2	Aconitate hydratase, mitochondrial	TCA
*XM_013086643*.1*	0.76	4.3E-15	-0.55	3.79E-07	KCNAB2	Voltage-gated potassium channel subunit beta-2	ion channel subunit, regulates other KCN [74]
*XM_005110731*.*2*	0.76	9.3E-15	-0.50	5.63E-06	SDHC	Succinate dehydrogenase cytochrome b560 subunit, mitochondrial	TCA and OXPHOS
*XM_005108990*.*2*	0.75	3.4E-14	-0.44	9.78E-05	ETFRF1	Electron transfer flavoprotein regulatory factor 1	regulator of the electron transfer flavoprotein [75]
*XM_005104950*.*2*	0.74	4.2E-14	-0.53	1.70E-06	KIF3A	Kinesin-like protein	anterograde transport [76]
*NM_001204727*.1*	0.74	9.7E-14	-0.52	2.29E-06	STAU2	Double-stranded RNA-binding protein Staufen homolog 2	transport of neuronal RNA from the cell body to dendrites [77]
*XM_013089873*.1**	0.74	1.3E-13	-0.61	1.08E-08	ITCH	E3 ubiquitin-protein ligase Itchy homolog	ROS defense [78], Involved in the negative regulation antiviral responses [79]
*XM_005103012*.2**	0.71	3.1E-12	-0.48	2.02E-05	PRDX5	Peroxiredoxin-5, mitochondrial	Antioxidant [80]

The RefSeq ID of each transcript is paired with a human protein ortholog gene symbol and name annotated by BLASTn mapping to the UNIPROT human proteome (UP000005640). The function of each ortholog is detailed in the final column. Correlation of transcript expression and chronological age, transcript-Age correlation (TAS), and significance (TAS p-value) are also listed. Transcripts are marked with “*” if they were among significantly downregulated transcripts in Kron et al 2020 [24] in one neuron type and with “**” if in both. All subsequent tables are organized identically. Common functions include TCA cycle, glycolysis, retrograde and anterograde transport, and calcium homeostasis. The module eigengene trend of this module was negatively correlated with age, indicating downregulation of these processes in aging.

#### 3.4.2 Pink

**Fig 5:**
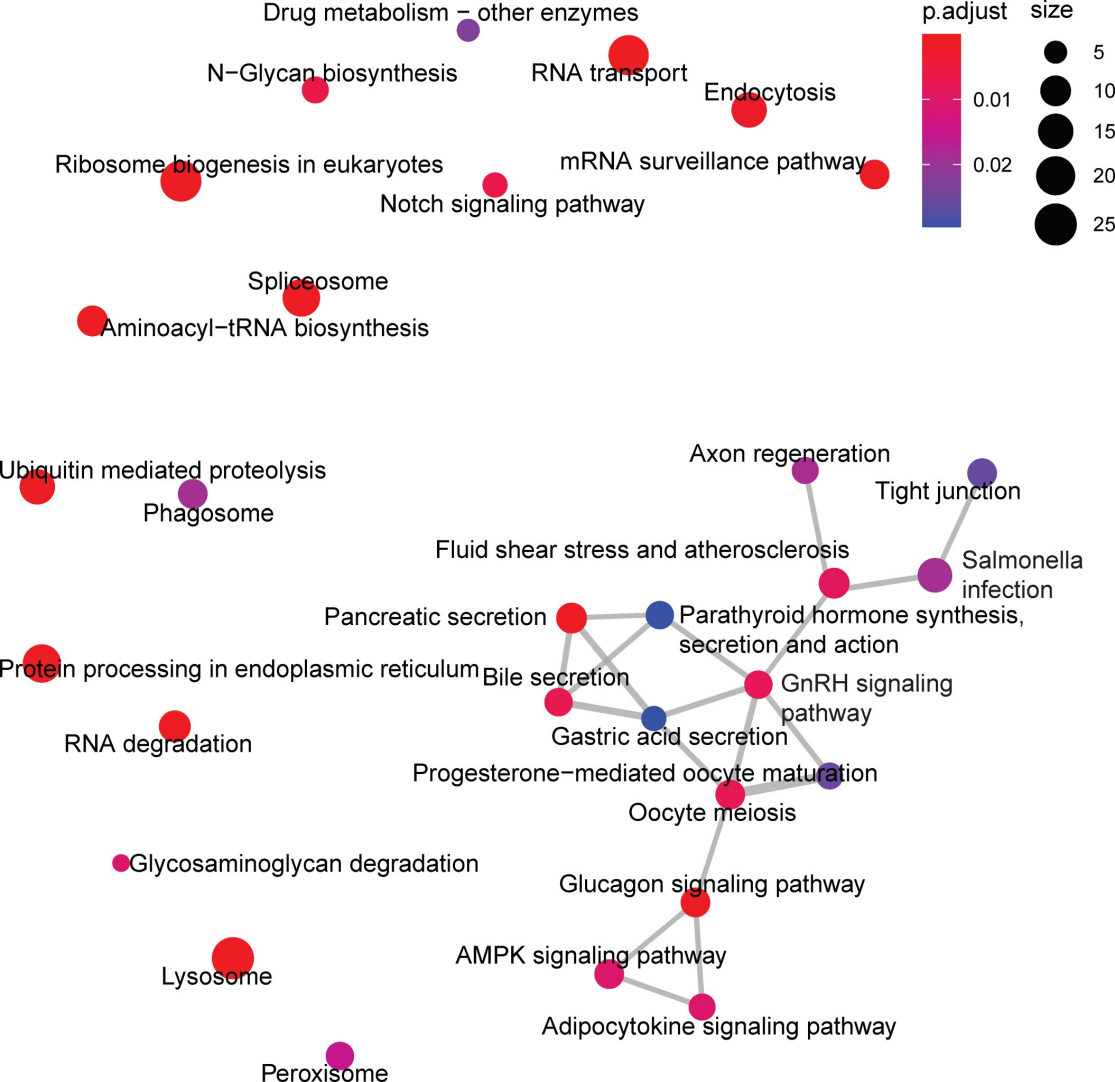
Enrichment map of Kyoto Encyclopedia of Genes and Genomes (KEGG) pathways for the *pink* consensus co-expression module. Symbol explanation as in Fig 4. The *Protein processing in the endoplasmic reticulum*, *Lysosome*, and *Ribosome biogenesis in eukaryotes* pathways are among the largest and most significantly enriched pathways. This module was positively correlated with age, suggesting these pathways are upregulated in aging.

The *pink* module, which was positively correlated with age, was significantly enriched for KEGG pathways related to translation, such as *ribosome biogenesis in eukaryotes* (ko03008) and *aminoactyl-tRNA biosynthesis* (ko00970), and proteostatic mechanisms, such as *lysosome* (ko04142), *protein processing in the endoplasmic reticulum* (ko04141), and *ubiquitin mediated proteolysis* (ko04120, Fig 5).

Investigation of individual transcripts with the highest module membership for the *pink* module similarly revealed several transcripts annotated to human orthologs involved in transcription, translation, and ribosome biogenesis, as well as modulation of innate immunity and NFkB signaling (Table 3).

**Table 3:** Selection of transcripts with highest correlation to transcript co-expression module eigengene (module membership, MM) in the *pink* consensus module identified in *Aplysia californica* sensory neurons by *WGCNA*.

RefSeq ID	MM	MM p value	TAS	TAS p value	Human Ortholog	Ortholog Name	Ortholog Function
XM_005111489.2**	0.95	1.8E-36	0.66	1.4E-10	**NFKBIA**	**NF-kappa-B inhibitor alpha**	NF-kappa-B inhibition [81], anti-inflammatory [82]
XM_005111747.2**!	0.93		0.74	6.8E-14	BIRC3	Baculoviral IAP repeat-containing protein 3	E3 ubiquitin-protein ligase, NF-kappa-B signaling regulation [83], innate immunity regulation [84]
XM_005106964.2*	0.84	4.1E-32	0.56	2.3E-20			
*XM_013081148.1*	0.78	3.15E-16	0.48	2.1E-05			
XM_005098591.2**	0.93	1.5E-31	0.68	5.6E-11	ZUP1	Zinc finger-containing ubiquitin peptidase 1	Deubiquitination, DNA damage, replication stress [85]
*XM_005098861.2*	0.92	1.2E-30	0.63	2.3E-09	UBC	Polyubiquitin-C (Fragment)	Ubiquitination [86]
*XM_005098862.2*	0.87	5.6E-23	0.53	1.7E-06			
XM_005105067.2**!	0.91	6.5E-29	0.65	7.3E-10	HERC4	Probable E3 ubiquitin-protein ligase HERC4	E3 ubiquitin-protein ligase [87]
XM_005112342.2**	0.91	6.6E-29	0.66	1.4E-10	ZNF343	Zinc finger protein 343	transcriptional regulation
XM_005108387.2**	0.91	2.1E-28	0.63	1.7E-09	ACP7	Acid phosphatase type 7	Iron transport, innate immunity, ROS generation [88]
XM_005092478.2*	0.90	7.5E-27	0.47	2.4E-05	INTS1	Integrator complex subunit 1	snRNA [89], eRNA [90], transcriptional attenuation [91,92]
XM_005097515.2**	0.90	9.3E-27	0.63	2.3E-09	GM2A	Ganglioside GM2 activator	Ganglioside metabolism [93]
XM_005111000.2**	0.90	1.3E-26	0.61	1.1E-08	GCN1	eIF-2-alpha kinase activator GCN1	Global translation repression, gene-specific mRNA translation [94]
XM_005090686.2**	0.89	5.2E-26	0.60	2.4E-08	SLC16A5	Monocarboxylate transporter 6	Glucose and lipid metabolism, possible immune regulation [95]
XM_005104555.2**	0.89	6.9E-26	0.59	3.5E-08	EFL1	Elongation factor-like GTPase 1	Ribosome biogenesis [96]
XM_005093568.2**	0.89	7.1E-26	0.53	1.3E-06	NAT10	RNA cytidine acetyltransferase	Ribosome biogenesis [97], E3 ubiquitin-protein ligase, cellular stress sensor [98], translation efficiency [99]
XM_005095929.2**	0.89	8.2E-26	0.58	7.5E-08	MOS	Proto-oncogene serine/threonine-protein kinase mos	Serine/threonine-protein kinase, MAPK pathway [100]
XM_005093424.2*!	0.89	1.8E-25	0.66	1.5E-10	BIRC7	Baculoviral IAP repeat-containing protein 7	E3 ubiquitin-protein ligase, apoptosis inhibitor [101]
XM_005094992.2*!	0.81	2.81E-18	0.66	1.9E-10			
XM_005102721.2*	0.89	2.0E-25	0.63	3.4E-09	GIMAP4	GTPase IMAP family member 4	Apoptosis [102], cytokine signaling [103]
XM_005106556.2**	0.88	6.1E-25	0.60	2.3E-08	ETF1	Eukaryotic peptide chain release factor subunit 1	Translation termination [104]
*XM_005093041.2*	0.88	1.3E-24	0.62	5.5E-09	RNASET2	Ribonuclease T2	Innate immunity [105], mtRNA degradation [106]
XM_005104568.2**	0.88	3.1E-24	0.61	1.0E-08	PLIN2	Perilipin-2	Lipid storage, ROS defense [107]
XM_013087467.1**	0.88	3.2E-24	0.60	1.7E-08	EIF4A2	Eukaryotic initiation factor 4A-II	Translation initiation [108], Translation inhibition [109]
XM_013087273.1**	0.87	7.7E-24	0.56	2.4E-07	HEATR1	HEAT repeat-containing protein 1	Ribosome biogenesis [110]
*XM_005101849.2*	0.87	8.5E-24	0.57	1.3E-07	EXOSC10	Exosome component 10	RNA metabolism [111]
XM_005099415.2**	0.87	1.1E-23	0.63	2.3E-09	DUSP7	Dual specificity protein phosphatase 7	MAPK pathway [112]
XM_005088796.2**!	0.87	1.2E-23	0.64	1.4E-09	IRF8	Interferon regulatory factor 8	Microglia activation and neuroinflammation [113]
XM_013084591.1**	0.87	1.6E-23	0.64	1.5E-09	PSAP	Prosaposin	Sphingolipid metabolism [114]
XM_013087976.1**	0.87	2.9E-23	0.60	1.9E-08	EEF2	Elongation factor 2	Translation [115]
XM_005097092.2**	0.86	6.1E-23	0.66	2.1E-10	CTSL	Cathepsin L1	Lysosomal protease [116], neuropeptide processing [117]
XM_005094650.2*!	0.86	6.8E-23	0.69	2.1E-11	CYLD	Ubiquitin carboxyl-terminal hydrolase CYLD	NF-kappa-B regulation, deubiquitination [118], Negative regulation of innate immunity [119]
XM_005090468.2**	0.86	9.1E-23	0.71	2.6E-12	PDE12	2’,5’-phosphodiesterase 12	Negative regulation of innate immunity
XM_005099805.2*	0.86	1.3E-22	0.49	9.2E-06	CASP3	Caspase-3	Apoptosis [120]
XM_013087138.1**	0.86	2.6E-22	0.50	6.1E-06	GRN	Granulins	Lysosome biogenesis and homeostasis [121]
XM_013083177.1**	0.85	6.4E-22	0.59	5.0E-08	SIGIRR	Single Ig IL-1-related receptor	Negative regulation of immune signaling [122]
XM_005110683.2**	0.84	2.0E-20	0.62	5.0E-09	FTH1	Ferritin heavy chain	Iron storage, ROS defense [123]
XM_013089050.1*	0.81	1.8E-18	0.56	3.2E-07	MAP3K8	Mitogen-activated protein kinase kinase kinase 8	MAPK signaling, NFkB signaling [124]
XM_005112843.2**!	0.81	2.2E-18	0.50	6.3E-06	RIOK1	Serine/threonine-protein kinase RIO1	Ribosome biogenesis [125], p38 MAPK innate immune response suppressor [126]
XM_005105539.2*	0.8	2.3E-17	0.45	6.9E-05	RIOK3	Serine/threonine-protein kinase RIO3	Ribosome biogenesis [127], INF signaling [128], NFkB inhibitor [129], innate immune response [130]
XM_005102490.2*	0.79	8.4E-17	0.60	1.6E-08	JKAMP	JNK1/MAPK8-associated membrane protein	MAPK signaling, inhibits MAPK8 [131]
XM_005092503.2**	0.78	2.3E-16	0.66	2.3E-10	TNIP1	TNFAIP3-interacting protein 1	NFkB inhibitor [132,133]
XM_013087081.2*	0.78	5.3E-16	0.48	2.15E-05	DUOX1	Dual Oxidase 1	ROS production [134]
*XM_013088029.1!*	0.69	1.3E-11	0.54	8.6E-07			

See Table 2 for description of organization. Transcripts are marked with “*” if they were among significantly upregulated transcripts in Kron et al 2020 [24] in one neuron type and with “**” if in both. Transcripts identified as orthologs to genes differentially expressed due to immune challenge in *C*. *gigas* are marked with a “!”in the first column. Common categories include ubiquitination, NFkB signaling, innate immunity, ribosome biogenesis, and regulation of transcription and translation. This module was positively correlated with age, suggesting these processes are upregulated in aging.

#### 3.4.3 Orange

The three KEGG pathways significantly enriched in the *orange* module, which was positively correlated with age, all participate in proteostasis, whether that is proper protein folding localized to the Endoplasmic Reticulum (ER) in the case of *protein processing in the endoplasmic reticulum* (ko04141) and *N-glycan biosynthesis* (ko00510), or in protein degradation, in the case of *lysosome* (ko04142). Transcripts with highest module membership were associated with ER stress or the endoplasmic reticulum associated protein degradation (ERAD) pathway (Table 4).

**Table 4:** Selection of transcripts with highest correlation to transcript co-expression module eigengene (module membership, MM) in the *orange* consensus module identified in *Aplysia californica* sensory neurons by *WGCNA*.

*Refseq ID*	MM	MM p value	TAS	TAS p value	Human Ortholog	Ortholog Name	Ortholog Function
*XM_005096841*.2*	0.93	6.27E-33	0.63	1.8E-09	**CREB3L3**	**Cyclic AMP-responsive element-binding protein 3-like protein 3**	ER stress response transcription factor, acute inflammation [135]
*NM_001204489*.1*	0.93	4.47E-32	0.52	2.0E-06	PSEN2	Presenilin-2	Endoprotease, Ca^2^+ homeostasis as ER leak channel [136], ER-Mitochondrial Ca^2^+ shuttle [137]
*XM_005101813*.2*	0.89	6.49E-26	0.56	2.5E-07	LGMN	Legumain	Endopeptidase [138]
*XM_013080474*.1*	0.89	7.70E-26	0.60	2.0E-08	EIF2AK3	Eukaryotic translation initiation factor 2-alpha kinase 3	Known as PERK, ER stress response [139]
*XM_005108642*.2*	0.87	2.24E-23	0.54	7.4E-07	MACO1	Macoilin	ER localized regulator of neuronal activity [140]
**XM_013081341*.*1**	0.86	7.08E-23	0.51	4.6E-06	DESI2	Deubiquitinase DESI2	Deubiquitinase [141]
*XM_013088003*.1**!	0.86	4.35E-22	0.71	2.7E-12	BIRC3	Baculoviral IAP repeat-containing protein 3	E3 ubiquitin-protein ligase, NF-kappa-B signaling regulation [83], innate immunity regulation [84]
*XM_005105526*.2**	0.85	1.13E-21	0.56	2.8E-07	ABCA3	ATP-binding cassette sub-family A member 3	Lipid transporter [142]
*XM_005113508*.1**	0.85	4.97E-21	0.57	1.0E-07	PSAP	Prosaposin	Sphingolipid metabolism [114]
**XM_013086889*.*1**	0.84	6.78E-21	0.45	6.2E-05	SPTLC2	Serine palmitoyltransferase 2	Sphingolipid de-novo synthesis
*XM_013079527*.1*	0.84	1.12E-20	0.55	5.8E-07	SUN1	SUN domain-containing protein 1	Neurogenesis and neuron and glial migration [143], telomere attachment [144]
*XM_005100110*.2*	0.83	6.91E-20	0.41	3.0E-04	MBOAT7	Lysophospholipid acyltransferase 7	Regulation of free polyunsaturated fatty acids levels [145,146]
*XM_013085224*.1**	0.83	1.34E-19	0.68	5.7E-11	VCAN	Versican core protein	Diverse roles [147], including inflammation [148]
*XM_005096173*.2**	0.83	1.71E-19	0.58	6.3E-08	BCL3	B-cell lymphoma 3 protein	Enhances or inhibits NFkB signaling depending on phosphorylation state [149,150]
*XM_005102600*.2*	0.82	1.24E-18	0.48	1.7E-05	SLC39A2	Zinc transporter ZIP2	Zinc transporter
*XM_013083287*.1*	0.81	4.10E-18	0.51	4.0E-06	ADGRG6	Adhesion G-protein coupled receptor G6	Schwann cell differentiation and myelination [151]
*XM_005101146*.2*	0.81	4.14E-18	0.57	1.7E-07	C16orf62	UPF0505 protein C16orf62	Cell surface recycling, including of signaling receptors [152]
**XM_005095917*.*2**	0.79	1.10E-16	0.36	1.8E-03	STT3B	Dolichyl-diphosphooligosaccharide—protein glycosyltransferase subunit STT3B	N-glycosylates unfolded proteins [153], plays role in ERAD [154]
**XM_005103010*.*2**	0.78	3.77E-16	0.45	7.5E-05	TXNDC16	Thioredoxin domain-containing protein 16	ERAD [155], humoral immune response [156]
*XM_005100158*.2*	0.78	7.92E-16	0.43	1.4E-04	UGGT1	UDP-glucose:glycoprotein glucosyltransferase 1	ER glycoprotein quality control [157]

See Table 2 for description of organization. Common functions include Endoplasmic Reticulum (ER) stress response, ER associated protein degradation (ERAD), sphingolipid metabolism, and immune regulation. This module was positively correlated with age, suggesting these process are upregulated in aging.

#### 3.4.4 Darkgreen

**Fig 6:**
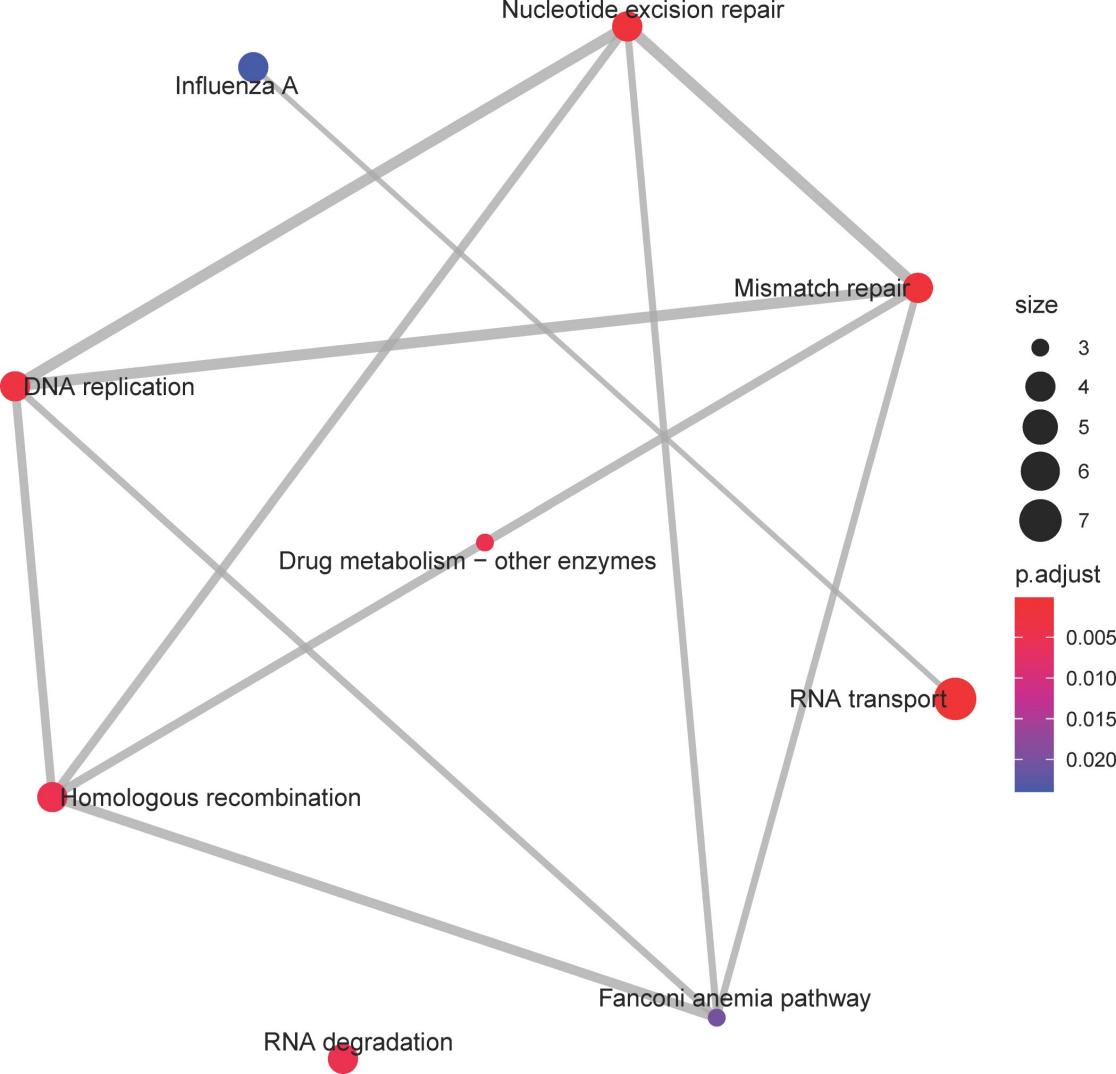
Enrichment map of Kyoto Encyclopedia of Genes and Genomes (KEGG) pathways for the *darkgreen* consensus co-expression module. Symbol explanation as in Fig 4. Several pathways dealing with nucleic acid metabolism, such as *DNA replication* and *RNA degradation* are significant in this pathway. The expression trend of this module increased linearly until age 10 months after which it stabilized, suggesting increasing activity of these pathways until a stable activity level is reach in old age.

For the *darkgreen* module, which exhibited an increasing eigengene expression trend until age 10 months after which the trend stabilized, KEGG enrichment analysis highlighted processes involved in nucleic acid metabolism, namely *DNA replication* (ko03030), *Nucleotide excision repair* (ko03420), and *mismatch repair* (ko03430) for DNA and *RNA transport* (ko03013) and *RNA degradation* (ko03018) for RNA (Fig 6).

Many of the transcripts with highest module membership were involved with DNA damage response, as well as formation of the nuclear pore, mRNA quality control and export, and immune signaling cascades such as the JAK/STAT cascade, NFkB signaling, and RIG-1 signaling (Table 5).

**Table 5:** Selection of transcripts with highest correlation to transcript co-expression module eigengene (module membership, MM) in the *darkgreen* consensus module identified in *Aplysia californica* sensory neurons by *WGCNA*.

Refseq ID	MM	MM p value	TAS	TAS P value	Human Ortholog	Ortholog Name	Ortholog Function
*XM_013082889*.*1*	0.96	9.81E-41	0.39	6.56E-04	RPA2	Replication protein A 32 kDa subunit	DNA replication [158], DNA damage repair [159,160]
*XM_005094195*.*2*	0.95	3.60E-37	0.51	4.61E-06	TRIM3	Tripartite motif-containing protein 3	E3 ubiquitin-protein ligase, negative regulator of inflammation [161–163], inhibits synaptic plasticity [164]
*XM_013086128*.*1*	0.94	2.71E-34	0.47	2.42E-05	TIPARP	Poly [ADP-ribose] polymerase	Inhibitor of AHR-dependent transcription [165], suppressor of INF due to AHR activation [166], activator of INF due to ROS [167]
*XM_005113357*.*2*!	0.93	7.35E-33	0.53	1.71E-06	SOCS2	Suppressor of cytokine signaling 2[160]	Inhibits JAK/STAT signaling, promotes neurite outgrowth [168], regulates cytokine signaling [169,170]
*XM_005092855*.*1*!	0.91	3.77E-28	0.46	3.57E-05			
*XM_005108083*.*2*!	0.85	4.32E-21	0.42	2.17E-04			
*XM_005092772*.*2*	0.93	1.77E-32	0.29	1.39E-02	ENDOG	Endonuclease G, mitochondrial	mitochondrial biogenenesis and homeostasis [171,172], apoptosis [173,174]
*XM_005107816*.2*	0.93	3.24E-32	0.52	2.29E-06	HENMT1	Small RNA 2’-O-methyltransferase	piRNA biogenesis [175]
*XM_005095515*.2*	0.93	3.35E-32	0.60	2.12E-08	ICE2	Little elongation complex subunit 2	snRNA transcription [176]
*XM_005101982*.*2*	0.93	1.01E-31	0.38	8.51E-04	LGALS4	Galectin-4	Reduced pro-inflammatory cytokine secretion [177], inhibits myelination [178]
*XM_013087261*.1*	0.92	3.26E-31	0.48	1.80E-05	GALC	Galactocerebrosidase	Lysosomal degradation of galactocerebrosides [179]
*XM_013084530*.*1*	0.92	1.26E-30	0.43	1.36E-04	SMARCD1	SWI/SNF-related matrix-associated actin-dependent regulator of chromatin subfamily D member 1	Transcription activation and repression via chromatin remodeling as part of SWI/SNF complex [180], immune regulation [181]
*XM_005102729*.*2*	0.92	5.39E-30	0.28	1.59E-02	WDR53	WD repeat-containing protein 53	unknown
*XM_005105962*.*2*	0.92	8.43E-30	0.46	4.99E-05	EPSTI1	Epithelial-stromal interaction protein 1	Macrophage differentiation [182]
*XM_005111022*.*2*	0.92	1.03E-29	0.52	2.03E-06	RBBP9	Putative hydrolase RBBP9	Inhibits TGF-beta growth-inhibition [183]
*XM_005101042*.*1*	0.91	1.34E-29	0.44	9.85E-05	RPA3	Replication protein A 14 kDa subunit	DNA damage repair [184]
*XM_005108381*.*2*	0.91	1.78E-29	0.43	1.39E-04	MOV10L1	RNA helicase Mov10l1	piRNA biogenesis [185]
*XM_013088805*.*1*	0.91	2.10E-29	0.47	3.26E-05	SLC16A14	Monocarboxylate transporter 14	Neuronal aromatic-amino-acid transporter [186]
*XM_005100427*.2*	0.91	3.91E-29	0.50	6.50E-06	NSMCE1	Non-structural maintenance of chromosomes element 1 homolog	E3 ubiquitin-protein ligase, DNA damage response, and iron homeostasis [187]
*XM_013084495*.*1*	0.91	5.14E-29	0.42	1.84E-04	DDX58	Probable ATP-dependent RNA helicase DDX58	RIG 1, antiviral immune receptor [188]
*XM_005091253*.2*	0.91	9.96E-29	0.56	2.70E-07	TENT4A	Terminal nucleotidyltransferase 4A	mRNA stability and quality control [189,190]
*XM_013090188*.*1*!	0.91	1.24E-28	0.56	2.35E-07	MPEG1	Macrophage-expressed gene 1 protein	Antibacterial protein, pore-forming protein, innate immunity [191]
*XM_005088804*.*2*	0.91	1.75E-28	0.44	1.00E-04	PLA2G16	HRAS-like suppressor 3	Phospholipid metabolism [192], sensor for sites of viral entry [193]
*XM_013082099*.*1*	0.90	1.24E-27	0.42	1.97E-04	NUP214	Nuclear pore complex protein Nup214	Nuclear pore formation and protein import into nucleus [194,195]
*XM_013088902*.*1*!	0.89	1.45E-26	0.50	8.27E-06	DIS3L2	DIS3-like exonuclease 2	Mediates degradation of polyuridylated RNAs [196], mRNA metabolism [197]
*XM_005089228*.*2*	0.89	2.04E-26	0.48	2.03E-05	SSUH2	Protein SSUH2 homolog	Odontogenesis, upstream of several transcriptional regulators [163]
*XM_005107110*.*2*	0.89	2.47E-26	0.43	1.52E-04	PARP14	Protein mono-ADP-ribosyltransferase PARP14	Suppresses IFN-gamma response, induces IL-4 response, counteracts PARP9 [198]
*XM_013081916*.*1*	0.89	6.38E-26	0.53	1.16E-06	NUP98	Nuclear pore complex protein Nup98-Nup96	Nuclear pore formation, gene expression regulation [199]
*XM_005106449*.2*!	0.89	1.87E-25	0.57	1.31E-07	ZRANB3	DNA annealing helicase and endonuclease ZRANB3	Rewinds DNA and maintains genome stability, replication stress response [200,201]
*XM_005106672*.*2*!	0.88	3.94E-25	0.44	1.18E-04	JAK2	Tyrosine-protein kinase JAK2	JAK/STAT signaling, interferon gamma response [202]
*XM_005106450*.*2*	0.88	5.72E-25	0.46	3.81E-05	BANF1	Barrier-to-autointegration factor	Chromatin organization and gene expression [203], blocks viral DNA replication [204]
*XM_013085923*.*1*	0.87	7.99E-24	0.42	2.17E-04	TREX2	Three prime repair exonuclease 2	DNA repair, mRNA export, transcription [205–207]
*XM_013080704*.1*	0.87	8.51E-24	0.52	2.95E-06	VRK1	Serine/threonine-protein kinase VRK1	Regulates BANF1 [208], activates ATF2 [209]
*XM_005107122*.*2*!	0.87	4.34E-23	0.35	2.44E-03	GLE1	Nucleoporin GLE1	Export of mRNA from nucleus [210]
*XM_005110865*.*2*	0.86	5.48E-22	0.41	2.70E-04	DXO	Decapping and exoribonuclease protein	Pre-mRNA quality control [211]
*XM_005113277*.*2*!	0.85	6.73E-22	0.28	1.77E-02	NUP88	Nuclear pore complex protein Nup88	Nuclear pore formation [212]
*XM_005099732*.*2*	0.85	9.14E-22	0.35	2.59E-03	MTPAP	Poly(A) RNA polymerase, mitochondrial	mtDNA stabilization [213,214], histone mRNA degradation [215]
*XM_013087644*.*1*	0.77	3.02E-15	0.39	7.51E-04	RPUSD3	Mitochondrial mRNA pseudouridine synthase RPUSD3	mtRNA translation, mitochondrial ribosome biogenesis via pseudouridylation [216,217]
*XM_005104572*.*2*	0.74	1.10E-13	0.41	2.66E-04	GIMAP1	GTPase IMAP family member 1	Immune cell development [218]
*XM_005109614*.*2*!	0.73	1.84E-13	0.30	9.16E-03	XIAP	E3 ubiquitin-protein ligase XIAP	E3 protein-ubiquitin ligase, apoptosis inhibitor [219], NFkB activation [220]
*XM_005109011*.*2*	0.72	5.24E-13	0.25	2.98E-02	STRA6	Receptor for retinol uptake STRA6	Retinol importer [221]

See Table 2 for description of organization. Common functions include nuclear pore formation, DNA damage repair, immune and inflammation signaling. The expression trend of this module increased linearly until age 10 months after which it stabilized, suggesting increasing activity of these in early age and stable, heightened activity in old age.

#### 3.4.5 Greenyellow

**Fig 7:**
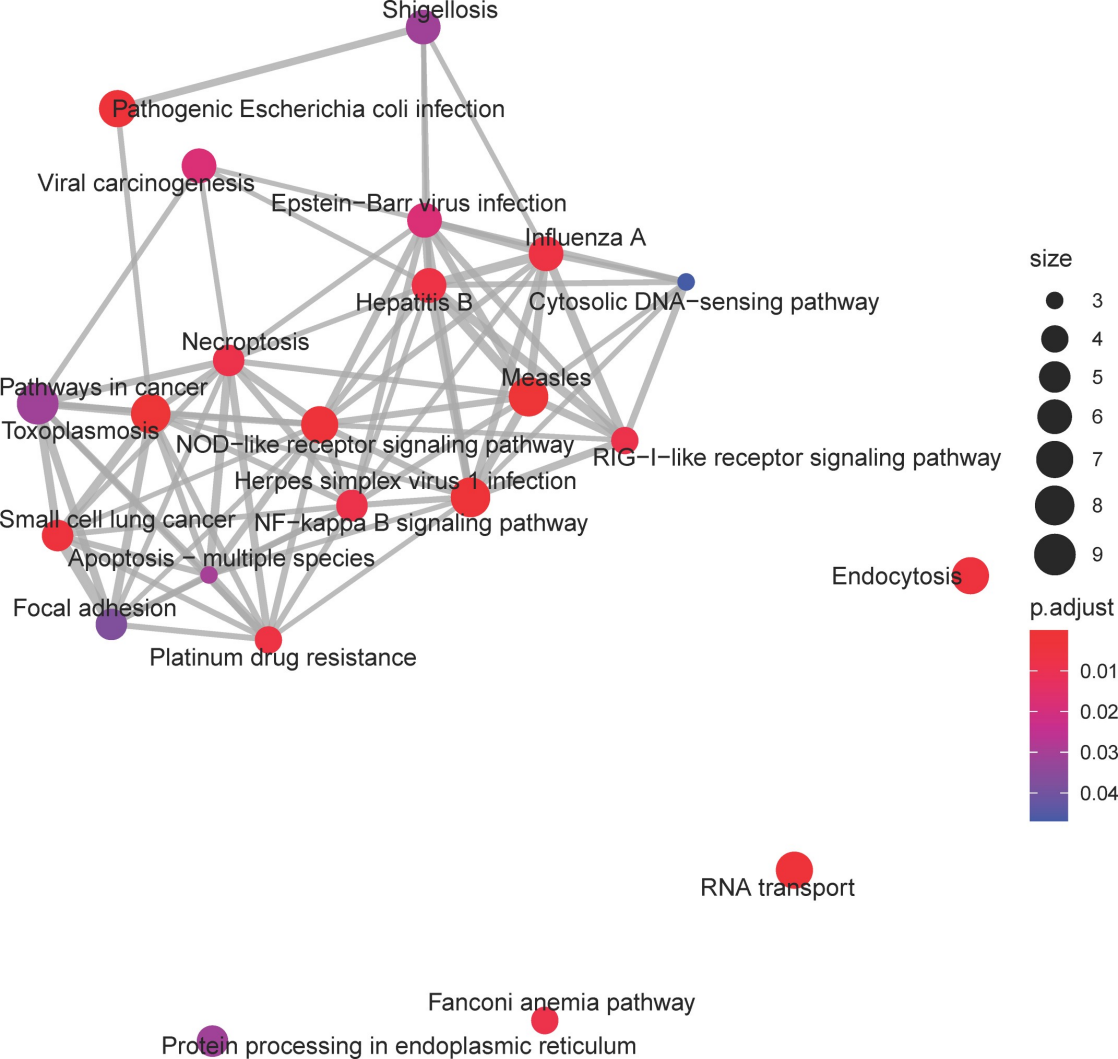
Enrichment map of KEGG pathways for the *greenyellow* consensus co-expression module. Symbol explanation as in Fig 4. Many of the significant pathways in this module have overlapping gene sets, many of which are associated with immune activation, such as *NOD-like receptor signaling*, *RIG-I-like receptor signaling*, *and NF-Kappa-B signaling pathway*. The increasing expression trend of this module’s eigengene throughout aging suggests consistently increased activation of these pathways in aging.

Pathways associated with viral infection and immune signaling dominated the KEGG enrichment results of the *greenyellow* module, which exhibited an increasing eigengene expression trend with age (Fig 7). Highly significant pathways included classical immune response associated pathways such as *NF-kappa-Beta signaling pathway* (ko04064), *RIG-1-like receptor signaling pathway* (ko04622), and *NOD-like receptor signaling* (ko04621). Transcripts with highest module membership in the *greenyellow* module mapped to human orthologs involved in the interferon and NFkB signaling pathways (Table 6).

**Table 6:** Selection of transcripts with highest correlation to transcript co-expression module eigengene (module membership, MM) in the *greenyellow* consensus module identified in *Aplysia californica* sensory neurons by *WGCNA*.

Refseq ID	MM	MM p value	TAS	TAS p value	Human Ortholog	Ortholog Name	Ortholog Function
*XM_005110768*.*2*!	0.95	1.8E-36	0.44	1.1E-04	**ZNFX1**	**NFX1-type zinc finger-containing protein 1**	Virus detection, IFN response [222], epigenetics [223]
*XM_005097053*.*2*!	0.92	6.8E-31	0.44	8.8E-05			
*XM_005105124*.*2*!	0.86	2.9E-22	0.53	1.4E-06			
*XM_013083625*.*1*	0.94	4.2E-35	0.42	1.9E-04	DTX3L	E3 ubiquitin-protein ligase DTX3L	E3 ubiquitin-protein ligase [224], DNA damage repair [225], INF response [226]
*XM_005102017*.*2*	0.94	1.8E-34	0.52	2.2E-06	SAMD9	Sterile alpha motif domain-containing protein 9	Antiviral stress response [227]
*XM_005090189*.2*	0.93	2.2E-32	0.45	5.9E-05	MRE11	Double-strand break repair protein	DNA damage repair [228], INF response [229]
*XM_005105514*.*2*	0.93	3.7E-32	0.46	4.5E-05	TRANK1	TPR and ankyrin repeat-containing protein 1	Interferon- stimulated gene [230]
*XM_013082259*.*1*!	0.93	1.1E-31	0.48	2.1E-05	TUT4	Terminal uridylyltransferase 4	mRNA decay [231], innate immunity [232]
*XM_005110839*.*2*	0.91	5.6E-29	0.47	2.5E-05	IL17RD	Interleukin-17 receptor D	ERK inhibitor [233], negative regulation of TLR signaling [234]
*XM_005099718*.*2*	0.90	7.9E-28	0.45	7.5E-05	VCPIP1	Deubiquitinating protein VCIP135	Deubiquitination [235]
*XM_005090454*.*2*	0.90	1.0E-27	0.56	3.4E-07	SACS	Sacsin	Chaperone [236], INF response in oyster
*XM_005106324*.2*	0.90	1.7E-27	0.60	2.6E-08	ZNF598	E3 ubiquitin-protein ligase ZNF598	E3 ubiquitin-protein ligase and Ribosome quality control [237], translation repression [238], Attenuation of innate immune response [239]
*XM_005093151*.*2*	0.90	2.7E-27	0.36	1.6E-03	TRIM2	Tripartite motif-containing protein 2	E3 ubiquitin-protein ligase [240], antiviral [241]
*XM_013081468*.1*	0.90	3.4E-27	0.52	2.0E-06	ASCC3	Activating signal cointegrator 1 complex subunit 3	DNA repair [242], NFkB, ATF-1, and SRF signaling [243]
*XM_005107621*.*2*!	0.90	4.2E-27	0.34	3.0E-03	CMTR1	Cap-specific mRNA (nucleoside-2’-O-)-methyltransferase 1	IFN signaling, antiviral state establishment [244]
*XM_013084387*.*1*!	0.90	6.5E-27	0.36	2.0E-03	STAT5B	Signal transducer and activator of transcription 5B	GH signaling [245], Il-2 cytokine signaling [246]
*XM_005095848*.*2*	0.90	1.3E-26	0.25	3.4E-02	ANK1	Ankyrin-1	Diverse, including protein localization to membranes [247]
*XM_005108543*.2*!	0.89	1.9E-26	0.63	2.2E-09	HERC4	Probable E3 ubiquitin-protein ligase HERC4	E3 ubiquitin-protein ligase [87]
*XM_005094842*.*2*!	0.89	4.7E-26	0.47	2.9E-05	IRF1	Interferon regulatory factor 1	IFN signaling [248]
*XM_005096699*.*2*	0.89	8.4E-26	0.31	7.1E-03	PARP12	Protein mono-ADP-ribosyltransferase PARP12	Interferon induced gens, negative regulation of translation, NFkB singaling [249]
*XM_005102776*.2*	0.88	4.5E-25	0.41	2.7E-04	DDX58	Probable ATP-dependent RNA helicase DDX58	RIG 1, antiviral immune receptor [188]
*XM_005104216*.*2*!	0.83	5.0E-20					
*XM_005098154*.*2*!	0.88	4.6E-25	0.55	5.7E-07	SMG1	Serine/threonine-protein kinase SMG1	Nonsense mediated mRNA decay [250], restriction of viral replication [251]
*XM_005101113*.*2*	0.88	7.1E-25	0.54	8.0E-07	ZC3HAV1L	Zinc finger CCCH-type antiviral protein 1-like	Same family as ZNFX1, unknown function [252]
*XM_013086154*.*1*	0.87	1.95E-23	0.32	5.2E-03	BIRC2	Baculoviral IAP repeat-containing protein 2	E3 ubiquitin-protein ligase, NF-kappa-B signaling regulation [83], innate immunity regulation [84]
*XM_013086147*.*1*	0.84	1.57E-20					
*XM_013089793*.*1*!	0.86	7.8E-23	0.43	1.7E-04	CASP8	Caspase-8	Apoptosis [253], inflammatory homeostasis via RIPK1 [254]
*XM_005111821*.*2*	0.85	1.6E-21	0.42	2.1E-04	HIST1H3A	Histone H3.1	Nucleosome formation, transcription regulation [255]
*XM_013079365*.*1*	0.83	6.6E-20	0.34	2.9E-03	TLR1	Toll-like receptor 1	Innate immune response [256,257]
*XM_013079178*.*1*!	0.83	7.2E-20	0.39	6.5E-04	IRF8	Interferon regulatory factor 8	Microglia activation and neuroinflammation [113]

See Table 2 for description of organization. Common functions include ubiquitination, interferon signaling (IFN), and inflammation. The roughly monotonic increasing trend of this module’s eigengene throughout aging suggests consistently increased activation of these processes in aging.

A full list of KEGG enrichment results can be found in Supplementary Datasheet **S1 Dataset**. Full transcript sets and their respective module membership values can be found in Supplementary Datasheet **S2 Dataset**.

### 3.5 Module enrichment for *C. gigas* immune response genes

BLAST annotation of Aplysia proteins to the *C*. *gigas* proteome and subsequent annotation resulted in 22,715 unique Aplysia transcripts mapped to 10,112 unique *C*. *gigas* proteins. Of these 22,715 transcripts, 7,359 were present in one of the identified co-expression modules. Among the 1,547 genes marked as exhibiting coherent regulation profiles following priming with poly(I·C)and viral challenge in Lafont et al (2020) [37], 697 were also present in Aplysia co-expression modules. Enrichment tests for each module revealed that the *greenyellow*, *darkgreen*, and *pink* modules were significantly enriched for *C*. *gigas* immune response genes (Fisher’s exact test, p < = 0.00385, Table 7). A full mapping of co-expression module genes to genes DE in Lafont et al (2020) [37] can be found in supplementary file **S3 Dataset**.

**Table 7:** Aplysia co-expression module enrichment for orthologs differentially expressed after immune priming and viral challenge in pacific oyster *Crassostrea gigas*.

	*Aplysia transcripts in C*. *gigas (m+n)*	Transcripts in LaFont (m)	Transcripts not in Lafont (n)	signif threshold (ɑ)	Bonferroni (ɑ`= ɑ/14)
	*7359*	697	6662	0.05	0.00385
**Module**	**Transcripts in module (k)**	**Module transcripts in LaFont (x)**	**Proportion (x/k)**	**p-value (p =** Σp(x-1:k)**)**	**Sig (**p<ɑ`)
*greenyellow*	224	45	0.20	4.37E-06	*
*darkgreen*	156	29	0.19	1.41E-03	*
*pink*	906	111	0.12	3.71E-03	*
*purple*	2203	206	0.09	6.72E-01	-
*blue*	1170	109	0.09	6.78E-01	-
*violet*	24	2	0.08	1.00E+00	-
*royalblue*	410	34	0.08	9.01E-01	-
*grey*	190	15	0.08	9.22E-01	-
*green*	1838	133	0.07	1.00E+00	-
*orange*	125	9	0.07	9.59E-01	-
*steelblue*	39	2	0.05	1.00E+00	-
*paleturquoise*	23	1	0.04	1.00E+00	-
*saddlebrown*	51	1	0.02	1.00E+00	-

Module enrichment for *C*. *gigas* immune genes in Aplysia co-expression modules was assessed via Fisher’s exact test. Of the 7359 Aplysia transcripts that were annotated as *C*. *gigas* orthologs and present in Aplysia co-expression modules, 697 were present in *C*. *gigas* (Lafont et al (2020) [37] m = 697, n = 6662). For each module, the number of transcripts in that module that were marked as *C*. *gigas* orthologs (k) and the proportion of those also present in Lafont et al (2020) [37] (x) were used to calculate the hypergeometric distribution in R. The p value was calculated as the sum of all probabilities at least as extreme as k (Σp(x-1:k) which was compared to a significance threshold of 0.05 with Bonferroni multiple test correction for 14 tests. Three modules (*greenyellow*, *darkgreen*, and *pink*) were identified as significantly enriched for *C*. *gigas* immune response orthologs.

Of the transcripts in the *greenyellow*, *darkgreen*, and *pink* modules, 17, 7, and 8 respectively exhibited a module membership greater than 0.8 for their respective modules and mapped to a *C*. *gigas* gene with a log 2 fold change of greater than or equal to 1 in response to immune priming and/or viral challenge in Lafont et al (2020) [37] (Table 8). Of those, transcripts from the *greenyellow* and *darkgreen* modules mapped primarily to *C*. *gigas* genes with putative viral function. Furthermore, the *greenyellow* transcripts mapped to C. gigas genes assigned by Lafont et al (2020) [37] primarily to the interferon-like and RIG-like receptor recognition pathways, while many *darkgreen* transcripts were assigned to the JAK/STAT signaling.

**Table 8:** Mapping between Aplysia transcripts with high module membership in the greenyellow, darkgreen, and pink modules and genes differentially expressed as a result of immune priming and viral exposure in *C. gigas*.

Module	Aplysia transcript	*C*. *gigas* Gene	Antimicrobial activity	Pathways	Human Ortholog	Ortholog Name
greenyellow	XM_005110768.2	CGI_10023396	virus	IFN-like pathway and RLR recognition	ZNFX1	NFX1-type zinc finger-containing protein 1
greenyellow	XM_013082259.1	CGI_10020126	V/B	other	TUT4	Terminal uridylyltransferase 4
greenyellow	XM_005097053.2	CGI_10003301	virus	IFN-like pathway and RLR recognition	ZNFX1	NFX1-type zinc finger-containing protein 1
greenyellow	XM_005107621.2	CGI_10013977	other	other	CMTR1	Cap-specific mRNA (nucleoside-2’-O-)-methyltransferase 1
greenyellow	XM_013084387.1	CGI_10028719	virus	JAK/STAT	STAT5B	Signal transducer and activator of transcription 5B
greenyellow	XM_005098154.2	CGI_10024989	V/B	signaling	SMG1	Serine/threonine-protein kinase SMG1 (Fragment)
greenyellow	XM_005101016.2	CGI_10021954	virus	IFN-like pathway and RLR recognition	DHX38	Pre-mRNA-splicing factor ATP-dependent RNA helicase PRP16
greenyellow	XM_013089793.1	CGI_10023960	virus	apoptosis	CASP8	Caspase-8
greenyellow	XM_005105124.2	CGI_10023396	virus	IFN-like pathway and RLR recognition	ZNFX1	NFX1-type zinc finger-containing protein 1
greenyellow	XM_005104216.2	CGI_10024392	virus	IFN-like pathway and RLR recognition	DDX58	Probable ATP-dependent RNA helicase DDX58
greenyellow	XM_013079178.1	CGI_10003270	virus	IFN-like pathway and RLR recognition	IRF8	Interferon regulatory factor 8 (Fragment)
greenyellow	XM_013085543.1	CGI_10018479	V/B	signaling	COL21A1	Collagen alpha-1(XXI) chain
greenyellow	XM_013081218.1	CGI_10021954	virus	IFN-like pathway and RLR recognition	DHX16	Pre-mRNA-splicing factor ATP-dependent RNA helicase DHX16
greenyellow	XM_005104310.2	CGI_10010459	virus	IFN-like pathway and RLR recognition	DDX58	Probable ATP-dependent RNA helicase DDX58
greenyellow	XM_005110422.2	CGI_10003270	virus	IFN-like pathway and RLR recognition	IRF8	Interferon regulatory factor 8
greenyellow	XM_005109293.2	CGI_10002009	other	other	ELF5	ETS-related transcription factor Elf-5
greenyellow	XM_005108818.2	CGI_10020752	virus	RNAi	DDX58	Probable ATP-dependent RNA helicase DDX58
darkgreen	XM_005113357.2	CGI_10019528	virus	JAK/STAT	SOCS2	Suppressor of cytokine signaling 2
darkgreen	XM_013090188.1	CGI_10002181	bacteria	other	MPEG1	Macrophage-expressed gene 1 protein
darkgreen	XM_005092855.1	CGI_10019528	virus	JAK/STAT	SOCS2	Suppressor of cytokine signaling 2
darkgreen	XM_013088902.1	CGI_10019733	virus	other	DIS3L2	DIS3-like exonuclease 2
darkgreen	XM_005108083.2	CGI_10019528	virus	JAK/STAT	SOCS2	Suppressor of cytokine signaling 2
darkgreen	XM_013091162.1	CGI_10028125	virus	other	RANBP2	E3 SUMO-protein ligase RanBP2
darkgreen	XM_013088005.1	CGI_10027619	virus	other	TYMP	Thymidine phosphorylase
pink	XM_005088796.2	CGI_10003270	virus	IFN-like pathway and RLR recognition	IRF8	Interferon regulatory factor 8 (Fragment)
pink	XM_005097229.2	CGI_10023430	other	other	FNIP2	Folliculin-interacting protein 2 (Fragment)
pink	XM_005110283.2	CGI_10026985	bacteria	other	PRSS12	Neurotrypsin
pink	XM_013081403.1	CGI_10026606	other	other	CBX4	E3 SUMO-protein ligase CBX4
pink	XM_005099789.2	CGI_10021954	virus	IFN-like pathway and RLR recognition	DHX16	Pre-mRNA-splicing factor ATP-dependent RNA helicase DHX16
pink	XM_013082526.1	CGI_10025856	virus	other	MSH2	DNA mismatch repair protein Msh2
pink	XM_005102215.2	CGI_10013829	V/B	other	ANGPTL6	Angiopoietin-related protein 6
pink	XM_005107413.2	CGI_10005182	virus	IFN-like pathway and RLR recognition	ADAR	Double-stranded RNA-specific adenosine deaminase (Fragment)

Transcripts (column 2) from the *greenyellow*, *darkgreen*, and *pink* (column 1) modules alongside the *C*. *gigas* gene to which they mapped (column 3) during BLAST comparison of Aplysia and *C*. *gigas* proteomes. Transcripts were selected if they exhibited module membership greater than or equal to 0.8 for their respective module and the *C*. *gigas* ortholog exhibited a log 2 fold change of at least 1 in Lafont et al (2020) [37] as a result of immune priming or virus exposure. Columns 4 and 5 list the antimicrobial function and pathway identified by Lafont et al (2020) [37] for each *C*. *gigas* gene. Columns 6 and 7 list the gene symbol and gene name for the human ortholog of each Aplysia transcript. A full mapping of Aplysia transcripts to *C*. *gigas* genes from Lafont et al (2020) [37] can be found in supplementary file **S3 Dataset**.

Of those *Crassostrea gigas* transcripts that exhibited a greater than two-fold increase in expression 10 days after treatment with a viral analog, 84 had clear orthologs in Aplysia. Of those 84 orthologous transcripts in Aplysia, only two exhibited a significant increase in expression in aging sensory neurons in our previous study: an IRF8 ortholog and an uncharacterized protein (S4 Dataset).

## 4 Discussion

While enrichment analysis and eigengene expression profiles suggested strong overlap with our previous study, several modules and enrichment results identified many facets to the transcriptional dynamics in aging of these sensory neurons not detected in our previous DEA study [24].

Enrichment analysis and eigengene expression trend of the *royalblue* module strongly resembles that observed in Kron et al (2020) [24] of expression clusters with decreasing expression trends in aging. Key enzymes in glycolysis such as *GPI*, *PGK1*, *PGAM2*, *GAPDH*, and *ENO1*, as well as *PDHB*, which is part of the pyruvate dehydrogenase complex that links glycolysis to the TCA cycle, were among the transcripts with the highest module membership in the *royalblue* module. Key TCA enzymes *DLST*, *IDH3G*, and *AOC2*, as well as two members of the succinate dehydrogenase complex, *SDHA* and *SDHC*, which function as a nexus between the TCA and OXPHOS, were also present. Downregulation of these transcripts suggests decreased TCA cycle activity, and decreased NADH generation for use in OXPHOS. The presence of orthologs to OXPHOS complex assembly proteins *SDHAF2*, *NDUFA2*, and *FMC1*, as well as electron transport chain regulator *ETRF1* among transcripts with high module membership may suggest further disruptions of OXPHOS at the complex level [41,71,73,75]. Mitochondria depleted of NADH exhibit impaired antioxidant capacity and increased ROS generation [258].

Several orthologs of major ROS detoxification enzymes, namely GPX4, SOD2, CAT, and *PRDX5* are present in the downregulated *royalblue* module [40,54,55,80]. The further presence of *PARK7*, *NXNL2*, and *ITCH*, which stimulate antioxidant activity, in this downregulated module suggest that the antioxidant system of these neurons is impaired in age [45,47,69,78]. The presence of ortholog mediators of mitochondrial fission-fusion dynamics such as *GDAP1* and *MFN2*, mediator of mitophagy *FUNDC1*, and promoter of mitochondrial biogenesis *GPS2*, which plays a role in mitochondrial stress signaling, suggests that maintenance of mitochondrial homeostasis is downregulated in aging [48,57,62,64,65]. Mitochondria act as key Ca^2+^ reservoirs and dysfunctional mitochondria can lead to perturbed Ca^2+^ dynamics. This is further suggested by downregulation of orthologs of *C1orf194* and *CALM2* which act to maintain Ca^2+^ homeostasis [50,53]. Homeostasis of Ca^2+^ is critical to the proper function of neurons suggesting that knock-on effects of energy metabolism impairment may have adverse effects on the proper functioning of these sensory neurons with aging. Similarly, the presence of two potassium channels orthologs, *KCNC2* and *KCNAB2*, a delayed rectifier K channel and a subunit of fast-inactivating A-type K channels, respectively, that repolarize neurons during firing and thus play important roles in membrane excitability, further suggests impaired neuronal function [63,74].

A facet of this module that was not captured in the expression clusters is the presence of many downregulated transcripts involved in cellular cargo transport. Orthologs of several proteins involved in retrograde transport, including *DCTN6*, *CCDC151*, and *EFCAB1*, and anterograde transport orthologs like *BLOC1S1* and *KIF3A*, suggest disruptions in communication from soma to synapse and vice versa [38,44,51,56,66,76,259]. Downregulation of *STAU2*, which plays a key role in transport of RNA from the cell body to dendrites for local translation, suggests disruption of transcription/translation events necessary for long term memory in these sensory neurons [77,260]. Other processes in cellular cargo transport, such as ER to Golgi transport, endocytosis and exocytosis are also represented by downregulated orthologs to *NAPG*, *EXOC2*, *SNX30*, *PDCD6*, *CHMP6*, *VPS26B*, and *SYT4* [52,60,68,72]. Proper vesicle transport and recycling are crucial for the signaling function of neurons, and disruptions in timing of these vesicle mediated events can impact neuronal function. The diversity of biological functions present in this module demonstrates the tight coupling of neuronal metabolism, transport of cellular cargo, and signaling.

While the *pink* module was identified as having an increasing eigengene expression trajectory and a largely a transcriptional and proteostatic character by KEGG enrichment analysis similar to several clusters in Kron et al (2020) [24], the transcripts with the highest module membership, including the hub gene *NFKBIA*, suggest inflammation plays a central role in this module. Many of these upregulated transcripts mapped to human genes known to be induced by NFkB, including *CSTL*, *BIRC3*, *FTH1*, *CYLD*, and the hub gene *NFKBIA* [261–266]. Furthermore, several of these orthologs activate or permit NFkB signaling, including *CSTL*, *BIRC3*, *GM2A*, and NAT10 [83,97,267–270]. The *pink* module also contains several dampeners of the NFkB signaling cascade and innate immunity, such as *NFKBIA*, *CYLD*, *PDE12*, *SIGIRR*, *RIOK1*, *RIOK3 JKAMP*, and *TNIP1*, likely to maintain homeostatic control and prevent over-inflammation [118,122,126,129–132,271,272]. While an upregulation of *NFKBIA* would seem to suggest a decrease in NFkB signaling, the degradation of *NKFBIA* is a key step in the release of NFkB, allowing translocation to the nucleus [272]. This may suggest that these neurons are upregulating NFKBIA to keep up with growing rates of *NKFBIA* degradation as a result of increased NFkB signaling [122].

The *pink* module also contains upregulated translation modulators. This includes genes that promote rRNA maturation and recruitment such as *EFL1*, *NAT10*, *RIOK1*, *RIOK3*, and *HEATR1* [96,97,110,125,127]. Although known for its role in ribosome biogenesis and translation efficiency [97,273,274], *NAT10* is also responsible for N^4^-acetylcytoside (N4A) driven INF and NFkB inflammatory signaling via *HMGB1* and *NLRP3* inflammasomes, perhaps playing a dual role in this module [270]. Chronic, low grade inflammation as a result of N4A accumulation due to consistent activity of *NAT10* may contribute to aging in these neurons.

Translation attenuating orthologs are also present in this module, such as *INTS1*, *GCN1*, and *EIF4A2* [91,92]. Under amino acid starvation conditions, *GCN1* activates *GCN2* which in turn phosphorylates *EIF4A*, which restricts translation [94,275–277]. However, this cascade has also been shown to be part of the antiviral response, specifically to prevent translation of viral RNAs [278,279]. Considering the preponderance of inflammatory genes in the *pink* module and the methodology that these animals were fed an *ad libitum* diet, the antiviral function of this cascade is the more likely here. Interestingly, this cascade also modulates synaptic plasticity and memory by inhibiting *CREB* in the hippocampus. Because *CREB* is essential for synaptic plasticity, increased *CREB* inhibition as a result of increased activity of the *GCN1*-*EIF4A* cascade during an inflammatory response may have knock-on effects that inhibit synaptic plasticity in these neurons [280].

Like the *pink* module, the *orange* module is similar to expression profile clusters found in Kron et al (2020) [24] as it exhibits increasing eigengene expression trajectory in age and classic signatures of ER stress. The hub transcript *CREB3L3* (*CREBH*) and the ortholog with fourth highest module membership *EIF2AK3* (*PERK*) are critical in the ER stress response cascade, while others like *UGGT1*, *STT3B* and *TNXDC16* are involved in ERAD [135,139,154,155,157]. Interestingly, several of the transcripts in the *pink* module with high module membership are involved in sphingolipid metabolism, such as PSAP and SPLITC2 [114,281,282]. Sphingolipids, particularly ceramides, play central roles in pro-inflammatory signaling and ER stress, suggesting this module also participates in pro-inflammatory signaling [283]. *SPLITC2* in particular has been shown to be upregulated by NFkB, suggesting NFkB signaling also plays a role in the *orange* module [281].

Several transcripts in the *orange* module regulate NFkB, such as *BCL3* and *BIRC3*, similar to the *pink* module [83,149,150]. In addition, upregulation of the *orange* module hub gene *CREB3L3* itself suggests neuro-inflammation as a central component of this module due to the role of this ortholog in the acute inflammatory response [135]. The similarities in the *pink* and *orange* modules suggest that each represents a different element of a proteostatic response to inflammation. Interestingly, the *darkgreen* module has a monotonic increasing eigengene expression trend early in the aging process and then stabilizes when the *orange* and *pink* modules enter their monotonic phase.

Many upregulated transcripts in the *darkgreen* module are orthologous to human genes associated with DNA damage response, including the hub transcript *RPA2*, *RPA3*, *NSMCE1*, *ZRANB3*, and *TREX2* suggesting mounting DNA damage with age in these neurons [160,184,187,201,205]. The presence of several upregulated transcripts orthologous to genes critical to the formation of the nuclear pore such as *NUP214*, *NUP98*, and *NUP88* as well as the stability and export of mRNA such as *TREX2*, *GLE1*, and *TENT4A*, and transcription and translation regulators like *SMARCD1* may suggest this module maintains a particular transcriptional program [180,189,190,206,207,210]. Interestingly, this module contains several upregulated orthologs known to inhibit inflammatory or immune signaling, such as *TRIM3*, *TIPARP*, *SOCS2*, *SMARCD1*, and *LGALS4*. This could suggest this module is either acting to suppress or modulate inflammatory signaling to prevent over-inflammation as seen in the *pink* and *orange* modules [162,166,170,177,181]. However, several other member orthologs exhibit pro-inflammatory functions, such as the hub transcript *RPA2* and *XIAP* which activate NFkB [220,284], viral RNA sensor and activator of the interferon response pathway DDX58/RIG-1 [285], and genes that support differentiation and maturation of immune cells such as *EPSTI1*, *RBBP9*, *SSUH2* and *GIMAP1* [163,182,183,218]. Aplysia immune hemocyte aggregates are known to play a pivotal role in neuron injury-associated inflammation and perhaps are similarly involved in the inflammatory response suggested by the orthologs present in the pink, orange, and *darkgreen* modules [286–289]. Curiously, *TIPARP* and *PARP14* function to suppress and activate different cytokine signaling cascades depending on context and could be counted among other previously listed groups [166,167,198]. Upregulation of orthologs to the aforementioned viral RNA sensor *RIG-1*, sensor of viral entry *PLA2G16*, antimicrobial peptide *MPEG1*, and blocker of viral DNA replication *BANF1* may suggest a role for this module in the detection of and initial response to viral infection in these neurons [191,193,204]. Interestingly, this may be further supported by the orthologs *HENMT1* and *MOV10L1* which mediate the formation of PIWI interacting RNAs (piRNA), noncoding RNAs that have antiviral activities in the innate immune systems of insects and possibly all eukaryotes [175,185,290,291]. The final module of interest, the *greenyellow* module, exhibits a monotonic increase in eigengene expression during the aging process, in parallel with the *darkgreen* module from age 6–9 months, and then parallel to the *pink* and *orange* modules thereafter.

Transcripts with highest module membership in the *greenyellow* module all represent orthologs to elements of the Interferon (IFN) Mediated response to RNA viruses. Several innate immune mobilized antiviral zinc finger protein orthologs are represented in this module, including *PARP12*, *ZC3HAVIL*, *TUT4*, and three orthologs of *ZNFX1*, one of which is the hub transcript [249,252,292,293]. Other transcripts are orthologs of interferon-stimulated genes (ISG) that stimulate or facilitate the expression of other ISG, including *ZNFX1*, *MRE11*, *DTX3L*, *CMTR1*, and *IRF1* [222,226,229,244,248]. Among these ISG orthologs are viral dsRNA sensors *ZNFX1* and *DDX58*/*RIG1* [222,285]. Other ISG orthologs upregulated in this module are IFN effector genes that have specific antiviral action: *SAMD9* triggers anti-viral granule formation, *TUT4* uridylates viral RNAs thus tagging them for degradation, *SMG1* restricts viral replication, and *TRIM2* prevents viral internalization [227,232,241,251]. The upregulation of *TLR1* and *STAT5b* orthologs in the *greenyellow* module, which cooperate with *STRA6* and *JAK2* from the *darkgreen*, capture elements of the pattern recognition signaling cascade needed to mobilize an immune response [246,256,257]. A proper immune response also requires modulators to prevent over-inflammation or to dampen ISG expression once the viral challenge has been dealt with to allow clearance of waste. Several such immune regulator orthologs are also upregulated in the *greenyellow* module, including *IL17RD* which exists as part of a larger family of proteins that regulate interleukin (IL) and Toll-like receptor (TLR) signaling, *ZNF598*, and *ASCC3* [234,239,243]. Whereas the *pink* module contains translation modulators, several of these upregulated transcripts are also orthologs of proteins known to have epigenetic and/or transcription attenuating effects, including *PARP12*, *ZNFX1*, and *H3*.*1*, possibly to mute global transcription in favor of anti-viral transcriptional programs [223,249,255].

Several of these transcripts also participate in NFkB signaling, promoting inflammation as part of the immune response. These include *IRF1* and *IRF8* which activate the Type-I Interferon response, *CASP8*, *ASCC3* which is indispensable for NFkB signaling, and *PARP12* which activates NFkB [243,249,254,294]. Ubiquitination dynamics are crucial for IFN and NFkB signaling [295], and many of the included upregulated transcripts are orthologs of genes that act as E3 ubiquitin-protein ligases such as *DTX3L*, *ZNF598*, *TRIM2*, and *HERC4*; as well as the Deubiquitinating enzymes *VCPIP1*. The multitude of orthologs involved in initiating the NFkB signaling cascade in response to viral infection in the *greenyellow* module may suggest this module may act upstream of the NFkB stimulated *pink* and *orange* modules.

While the discussed results appear to present a convincing picture of the function of these co-expression networks, it is important to approach these inferences with a degree of caution. Genes marked as orthologous by statistical means, such as BLAST or ghostKOALA, do not always perform the same functions in the target species as they do in the annotated species. Very few of these transcripts discussed have been independently investigated in Aplysia and their functions in Aplysia neurons and associated cells are not known. While several orthologs are generally understood to perform conserved roles across genera and their inferred function can be reasonably assumed, such as those involved in glycolysis or the TCA cycle, others involved in more idiosyncratic systems such as the immune response may have a large degree of divergence, especially when comparing the complex immune system of vertebrates such as human to that of evolutionarily distant mollusks.

Although the function of the orthologs discussed were characterized in human, mouse, *C*. *elegans*, and/or Drosophila, similar expression patterns to those captured by the *pink*, *orange*, *darkgreen*, and *greenyellow* modules have also been documented in other mollusks. Both *Cathepsin L* and *MPEG1* orthologs have been shown to take part in the innate immune response of two species of abalone [296–298]. Immune challenge in oysters with viral RNA also resulted in upregulation of common NFkB and JAK/STAT signaling components observed in *darkgreen* and *greenyellow* modules, including orthologs to *ZNFX1*, *Sacsin*, and *MPEG1* along with various IAP, *TRIM*, caspases, and IRF proteins *[37]*. To assess to what degree the transcripts inferred to play a role in the neural immune and inflammatory response via annotation to human orthologs represent a bonafide immune response, we further compared our module transcript sets to the transcriptional signature of an acute immune response to immune priming and RNA virus exposure in another mollusk, the Pacific oyster *Crassostrea gigas* [37]. The *pink*, *greenyellow*, and *darkgreen* modules were significantly enriched for orthologs to putative *C*. *gigas* immune response genes, supporting the notion that these modules do indeed represent a component of the Aplysia immune response. Indeed, several DE genes in the *C*. *gigas* immune response were orthologs of transcripts with high module membership in the *pink*, *darkgreen*, and/or *greenyellow* modules, such as the aforementioned orthologs of HERK4, BIRC3, IRF1, IRF8, MPEG1, DDX58/RIG-I, and most importantly all three orthologs of ZNFX1, including the *greenyellow* hub gene. Furthermore, several more transcripts, although not identified as orthologs of genes demonstrated to be DE in Lafont et al (2020) [37], do still map to the same human ortholog as genes identified in Lafont et al (2020) [37], such as *sacsin*. However, when comparing the list of significantly differentially expressed genes as a result of poly(I·C) in *C*. *gigas* to Aplysia orthologous genes significantly upregulated in sensory neuron aging as reported in our previous study, only two orthologs are shared. This suggest that, while the *greenyellow* and *darkreen* modules may capture an immune transcriptional program, viral infection and associated immune response are not drivers of transcriptional change in Aplysia sensory neurons. Moreover, this lack of correlation with oyster genes chronically upregulated after exposure to a virus analog indicates the need for caution in interpreting changes in module expression. Even when specific modules that show an increase in expression with aging include transcript members with anti-inflammatory or antiviral functions, these specific genes may not individually exhibit age-related expression changes. On the other hand, a majority of transcripts with high module membership in the *royalblue*, *pink*, and *orange* modules were identified as differentially expressed in our previous study, suggesting inflammation may result in proteostatic stress and mitochondrial dysfunction rather than infection.

Several transcripts from the *royalblue* module interact with the *pink* and *orange* modules, primarily as inhibitors. *MTFS2* specifically inhibits the activity of *EIF2AK3* from the *orange* module, which functions to maintain mitochondrial morphology, Ca^2+^ homeostasis, and limit ROS production [299]. *ITCH*, via its interaction with *TXNIP*, promotes an antioxidant state, prevents pro-inflammatory signaling through ROS and NFkB, and prevents *TXNIP* from inhibiting glycolysis [300,301]. Similarly, *PARK7* also inhibits NFkB signaling and the astrocyte inflammatory response [302,303]. Finally, GPS2 also exhibits strong anti-inflammatory activity [304,305]. Conversely, *PDE12* from the *pink* module is known to suppresses mitochondrial translation and contribute to respiratory incompetence [306]. Perhaps the eigengene expression perturbation at 9 months of age common to these expression modules may represent a transition from a state of healthy metabolic activity and antioxidant capacity exemplified by the transcripts of the *royalblue* module towards an aged state typified by inflammation and protein dyshomeostasis suggested by the transcript sets of the *pink* and *orange* modules. The role of ROS represents a potentially interesting link between these modules.

Decreased activity of mitochondrial homeostasis and metabolism mechanism suggested by the *royalblue* module suggest mitochondrial dysfunction with age, a well-known source of ROS [307]. Furthermore, downregulation of several antioxidant proteins in the *royalblue* module would suggest decreased capacity for ROS defense. Overexpression of SOD2 and GPX have been demonstrated to inhibit NFkB activation [308,309], thus the downregulation of these and other antioxidants in the *royalblue* module may in fact potentiate NFkB activation as suggested by the *pink* and *orange* modules via increased ROS levels. While the role of ROS in NFkB signaling is complex and cell type specific, prolonged oxidative stress has been shown to increased NFkB signaling and contribute to a pro-inflammatory state [310,311]. Upregulation of FTH by NFkB, as seen in the *pink* module, has also been suggested to be a major component of the NFkB derived antioxidant response against high H_2_O_2_ levels during chronic inflammation and oxidative stress [312]. Furthermore, ER stress resulting from increases in misfolded proteins due to oxidative stress, signatures of which were identified in our previous study and are also suggested by the *orange* module, has been shown to activate NFkB as well. Specifically, the activity of PERK, which is among the top orthologs in the *orange* module, plays a crucial role in activation of NFkB during ER stress [313]. In addition to downregulation of antioxidants, co-temporal upregulation of ROS producers like the two *DUOX1* orthologs in the *pink* module suggest an increased in ROS, [88,134].

Indeed, chronic, low-grade inflammation has been suggested to be both cause and consequence of mitochondrial dysfunction and resulting metabolic impairment in neuronal aging [314–316]. These data suggest that chronic inflammation contributes to an increasingly oxidative environment in these neurons, exacerbating mitochondrial dysfunction that is known to occur in aging.

## Supporting information

S1 Fig Scale free topology and mean connectivity calculated for expression data from PVC and BSC sensory neurons. For each cutout, the scale free topology model fit (A), median connectivity (B), mean connectivity (C), and max connectivity (D) of a co-expression matrix (y-axis) at a selected soft threshold power (x-axis) is plotted for both BSC (black) and PVC (Red). A soft power of 16 was selected for both sensory neuron types to achieve a scale free topology fit above 0.9 and to minimize the mean connectivity.
(TIF)

S2 Fig Hierarchical cluster of consensus co-expression module eigengenes derived from expression data from PVC and BSC sensory neuron clusters. Module eigengene names are arbitrarily assigned. Red horizontal line represents 0.25 branch height threshold for similarity, below which modules are merged.
(TIF)

S3 Fig Hierarchical cluster of consensus expression data from PVC and BSC sensory neuron clusters. Colored bars at the bottom represent module assignments for each transcript. Module colors are arbitrarily assigned. The top color set represents the module assignment before merging similar of similar modules, while bottom bar represents module assignment post merge (see **S2 Fig**). In total, 13 co-expression modules were identified.
(TIF)

S4 Fig Consensus co-expression module eigengene correlation with external phenotype calculated individually for constituent sensory neuron types (PVC and BSC sensory neurons). Each cell of the heatmap represents the correlation between a module eigengene (row) and phenotype (column). The top number in a cell is the value of Pearson correlation between two eigengenes. The p-value significance of module-trait correlation is the bottom number in each cell in parentheses. The phenotypes are animal weight at sacrifice (weight), latency of two reflex behaviors described in Greer et al 2018: Tail withdrawal reflex time (TWRT) and time to right (TTR), and chronological age of the animal in months at sacrifice (Age). Correlations for age are similar between sensory neuron types for the *royalblue*, *saddlebrown*, *greenyellow*, *orange*, *pink*, and *darkgreen* modules.
(TIF)

S1 Table Software. All Software and respective versions used for RNA sequencing read quality control and quality assurance, mapping and abundance estimation, and downstream analysis.
(DOCX)

S2 Table Transcript set overlap between co-expression modules (modules) and transcript expression profile clusters (clusters) from Kron et al (2020) [24]. Each cell represents the number of transcripts shared between respective module (row) and cluster (column). The “n” column represents the number of transcripts in a given module, and the “n” row represents the number of transcripts in a given cluster. Values in the “Sum” columns are row sums, e.g. the total number of transcripts in each respective module also present in the clusters. The “% of module” columns represent percentage values calculated by dividing the “Sum” columns by the total number of transcripts in a cluster set (1106 for B clusters, and 1198 for P clusters). Values in the “Sum” row are column sums, e.g. the total number of transcripts in each respective cluster that are also present in the modules. The “cluster %” row represent percentage values calculated by dividing the “Sum” row by the total number of transcripts among all modules (10012).
(DOCX)

S1 File R session. All R packages and respective versions used for clustering, analysis, and visualization of RNA sequencing transcript abundances.
(TXT)

S1 Dataset Full module KEGG enrichment results. Full results for all modules of KEGG enrichment of co-expression modules using clusterProfiler.
(XLSX)

S2 Dataset Full module membership results. Per module module-membership values and significance for all transcripts assigned to each module.
(XLSX)

S3 Dataset Module transcript overlap with genes DE in C. gigas immune response. This file contains the results of mapping *Aplysia californica* transcript IDs to *Crassostrea gigas* gene IDs for transcripts in Aplysia age associated co-expression modules that mapped to C. gigas genes identified as DE in immune priming and viral challenge by Laont et al 2020.
(XLSX)

S4 Dataset Evaluation of shared, upregulated orthologs in *C*. *gigas* immune response and Aplysia sensory neuron aging. This file contains a list of genes upregulated genes in *C*. *gigas* immune respone to viral analogue poly(IC) as reported in Lafont et al. 2020 [37] that have orthologs with evalue of >1E-20 in Aplysia. Aplysia orthologs are annotated with transcript, protein, and gene RefSeq identifiers as well as neuron type-specific adjusted pvalues, expression cluster assignment, and expression cluster direction reported in our previous publication. Only two Aplysia orthologs were identified as significantly upregulated in aging sensory neurons.
(XLSX)
