# Supplementary figures and images for: Co-expression analysis identifies neuro-inflammation as a driver of sensory neuron aging in Aplysia californica
Source: PLoS One. 2021 Jun 11;16(6):e0252647. doi: 10.1371/journal.pone.0252647 (PMC8195618; doi:10.1371/journal.pone.0252647)

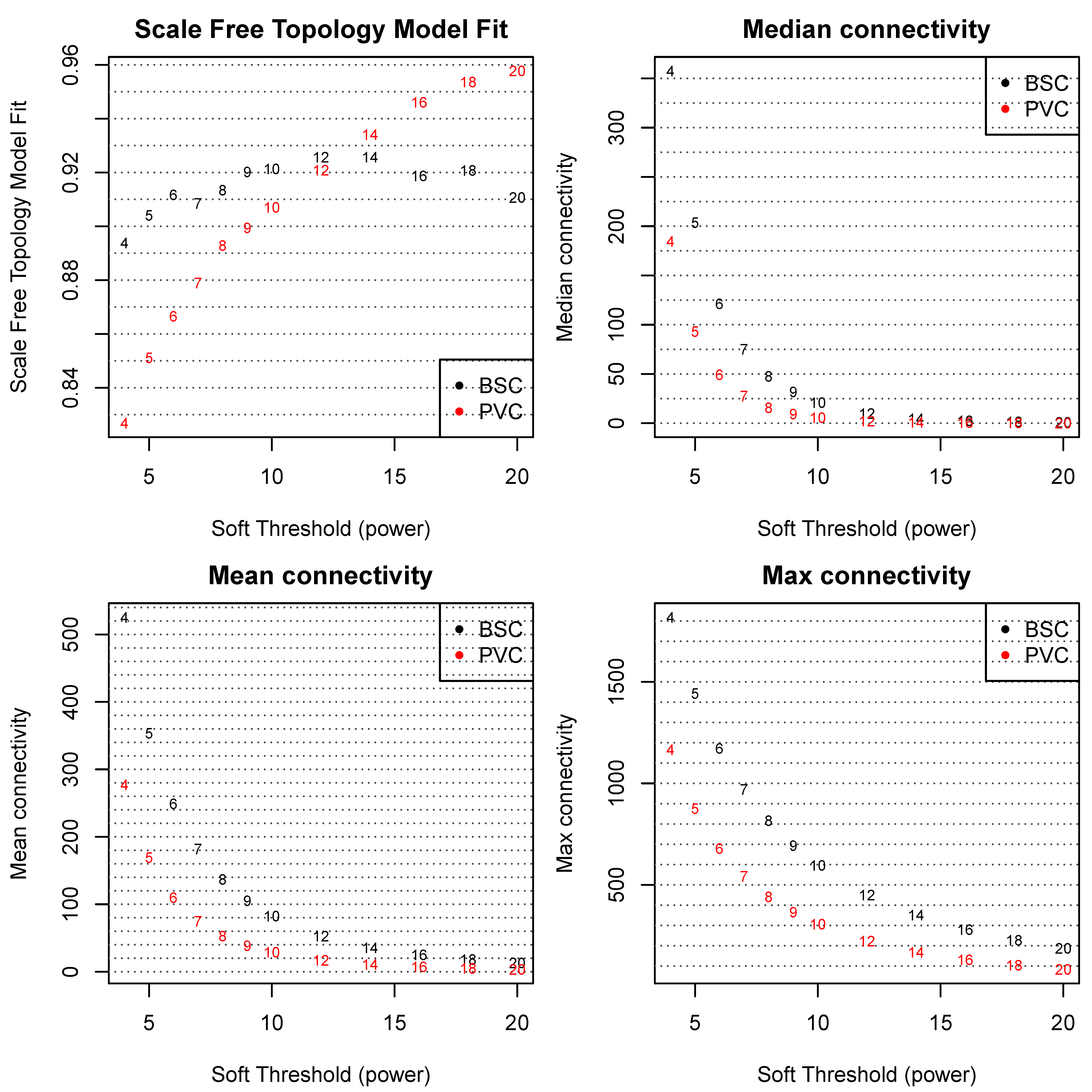

Supplement: S1 Fig — For each cutout, the scale free topology model fit (A), median connectivity (B), mean connectivity (C), and max connectivity (D) of a co-expression matrix (y-axis) at a selected soft threshold power (x-axis) is plotted for both BSC (black) and PVC (Red). A soft power of 16 was selected for both sensory neuron types to achieve a scale free topology fit above 0.9 and to minimize the mean connectivity. (TIF) [file pone.0252647.s001.tif]

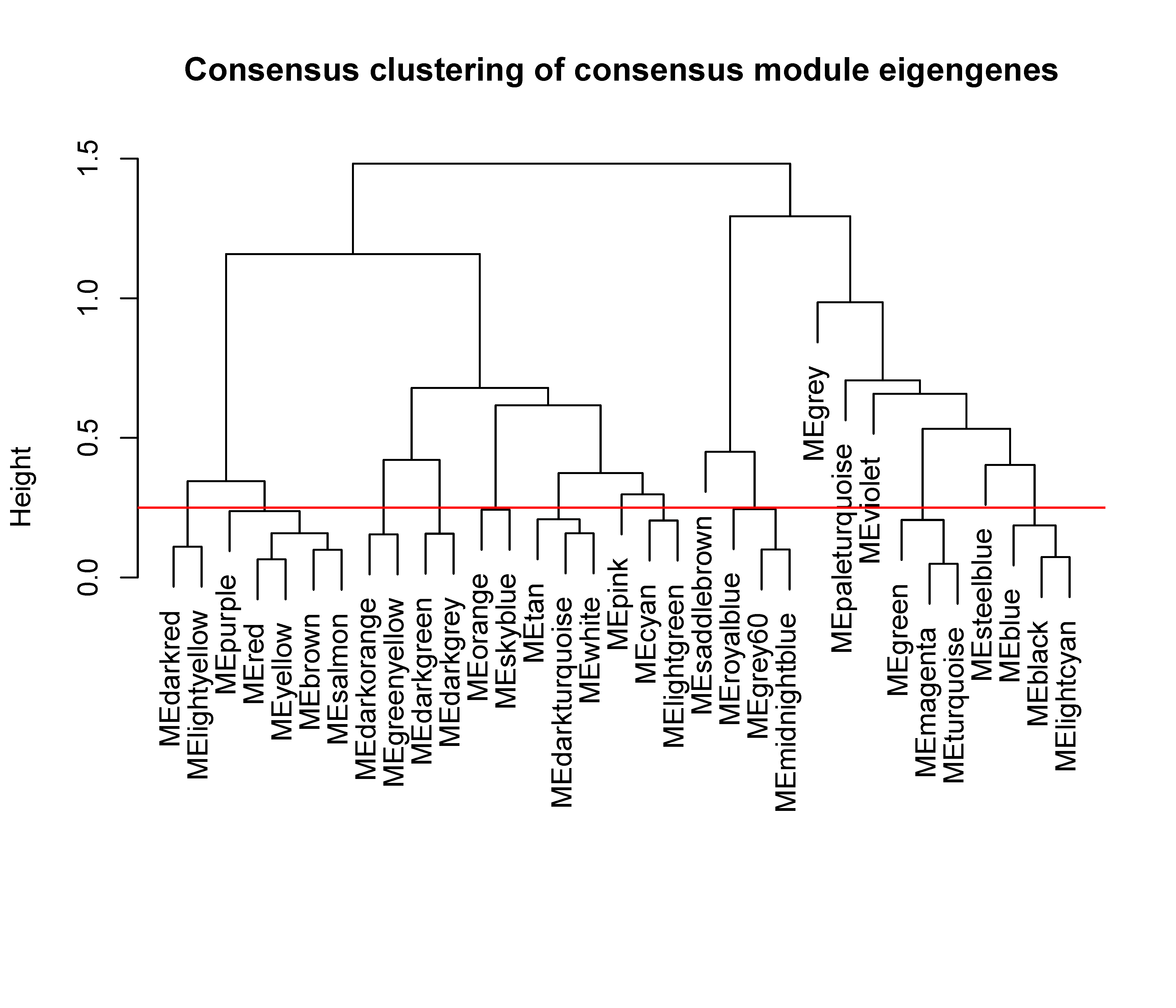

Supplement: S2 Fig — Module eigengene names are arbitrarily assigned. Red horizontal line represents 0.25 branch height threshold for similarity, below which modules are merged. (TIF) [file pone.0252647.s002.tif]

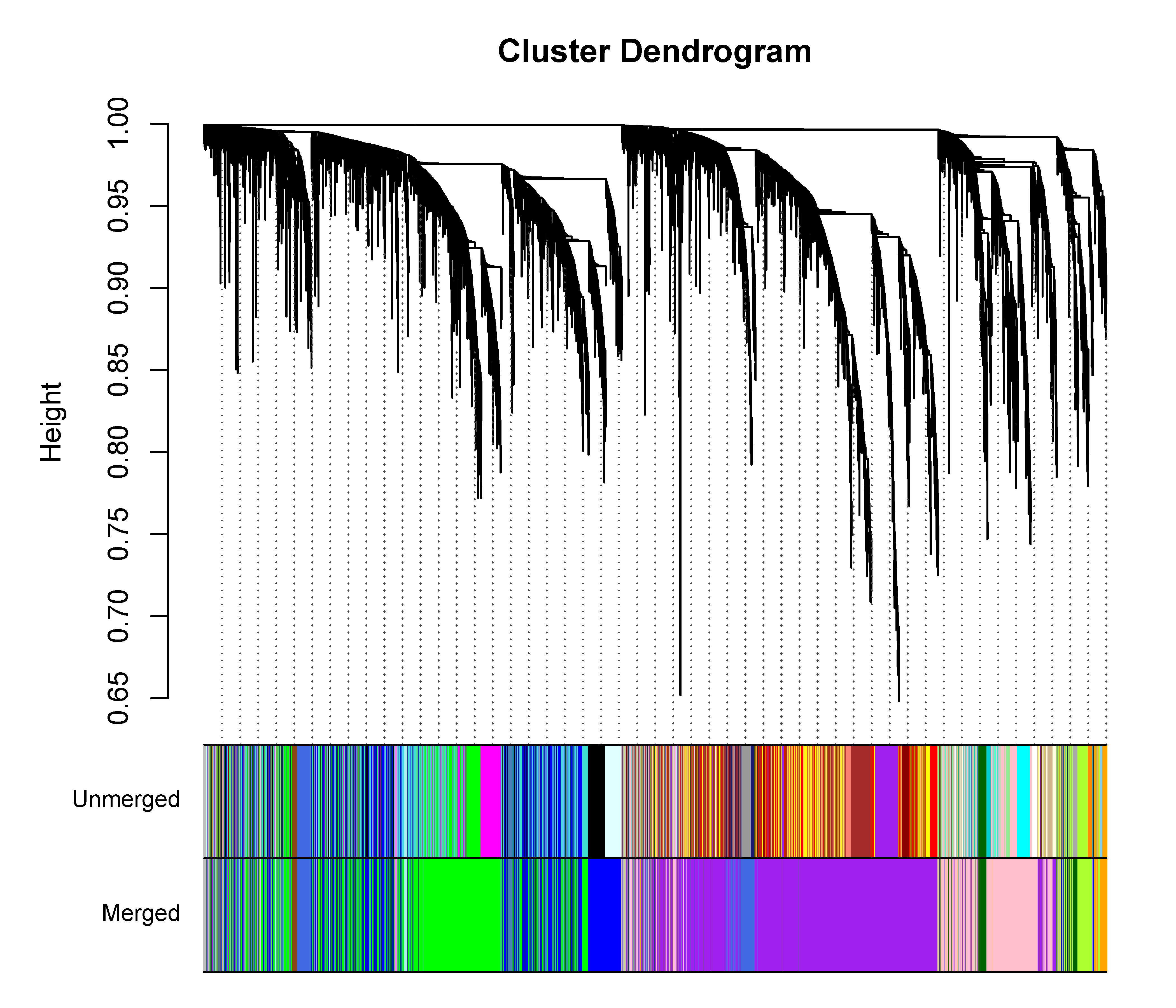

Supplement: S3 Fig — Colored bars at the bottom represent module assignments for each transcript. Module colors are arbitrarily assigned. The top color set represents the module assignment before merging similar of similar modules, while bottom bar represents module assignment post merge (see S2 Fig). In total, 13 co-expression modules were identified. (TIF) [file pone.0252647.s003.tif]

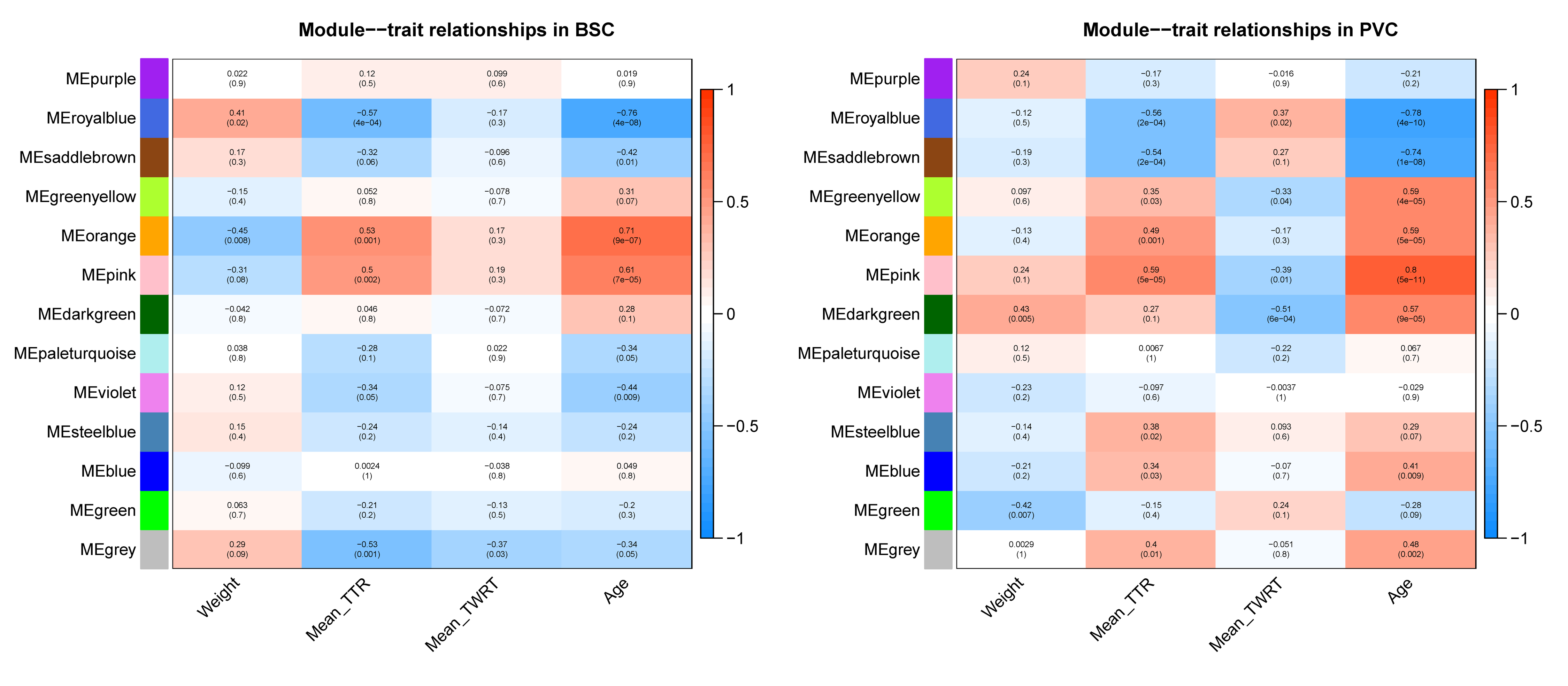

Supplement: S4 Fig — Each cell of the heatmap represents the correlation between a module eigengene (row) and phenotype (column). The top number in a cell is the value of Pearson correlation between two eigengenes. The p-value significance of module-trait correlation is the bottom number in each cell in parentheses. The phenotypes are animal weight at sacrifice (weight), latency of two reflex behaviors described in Greer et al 2018: Tail withdrawal reflex time (TWRT) and time to right (TTR), and chronological age of the animal in months at sacrifice (Age). Correlations for age are similar between sensory neuron types for the royalblue, saddlebrown, greenyellow, orange, pink, and darkgreen modules. (TIF) [file pone.0252647.s004.tif]
