# Supplementary material for: Co-expression analysis identifies neuro-inflammation as a driver of sensory neuron aging in Aplysia californica
Source: PLoS One. 2021 Jun 11;16(6):e0252647. doi: 10.1371/journal.pone.0252647 (PMC8195618; doi:10.1371/journal.pone.0252647)
Supplement: S1 Table — All Software and respective versions used for RNA sequencing read quality control and quality assurance, mapping and abundance estimation, and downstream analysis. (DOCX) [file pone.0252647.s005.docx]

**S1 Table. Software.**

| Software | Version | link |
| --- | --- | --- |
| FastQC | 0.10.1 | https://www.bioinformatics.babraham.ac.uk/projects/fastqc/ |
| BBTools | 37.90 | https://jgi.doe.gov/data-and-tools/bbtools/ |
| Salmon | 0.11.2 | https://combine-lab.github.io/salmon/ |
| R | 3.6.0 | https://www.r-project.org/ |
| RStudio | 1.2.1335 | https://rstudio.com/ |

All Software and respective versions used for RNA sequencing read quality control and quality assurance, mapping and abundance estimation, and downstream analysis.
