# Supplementary material for: Co-expression analysis identifies neuro-inflammation as a driver of sensory neuron aging in Aplysia californica
Source: PLoS One. 2021 Jun 11;16(6):e0252647. doi: 10.1371/journal.pone.0252647 (PMC8195618; doi:10.1371/journal.pone.0252647)
Supplement: S2 Table — Each cell represents the number of transcripts shared between respective module (row) and cluster (column). The “n” column represents the number of transcripts in a given module, and the “n” row represents the number of transcripts in a given cluster. Values in the “Sum” columns are row sums, e.g. the total number of transcripts in each respective module also present in the clusters. The “% of module” columns represent percentage values calculated by dividing the “Sum” columns by the total number of transcripts in a cluster set (1106 for B clusters, and 1198 for P clusters). Values in the “Sum” row are column sums, e.g. the total number of transcripts in each respective cluster that are also present in the modules. The “cluster %” row represent percentage values calculated by dividing the “Sum” row by the total number of transcripts among all modules (10012). (DOCX) [file pone.0252647.s006.docx]

**S2 Table.** **Transcript set overlap between co-expression modules (modules) and transcript expression profile clusters (clusters) from Kron et al 2020.**

|  | cluster 🡪 | B1 | B2 | B3 | B4 | B Sum | % of  module  ↓ | P1 | P2 | P3 | P4 | P5 | P Sum | % of module  ↓ |
| --- | --- | --- | --- | --- | --- | --- | --- | --- | --- | --- | --- | --- | --- | --- |
| Module ↓ | n , n | 54 | 440 | 449 | 163 | 1106 |  | 94 | 201 | 206 | 621 | 76 | 1198 |  |
| orange | 166 | 13 | 38 | 0 | 0 | 51 | 31% | 1 | 12 | 9 | 0 | 0 | 22 | 13% |
| pink | 1255 | 16 | 268 | 0 | 0 | 284 | 23% | 67 | 101 | 83 | 0 | 0 | 251 | 20% |
| greenyellow | 329 | 1 | 2 | 0 | 0 | 3 | 1% | 5 | 2 | 22 | 0 | 0 | 29 | 9% |
| darkgreen | 225 | 0 | 1 | 0 | 0 | 1 | 0% | 4 | 1 | 17 | 0 | 0 | 22 | 10% |
| purple | 3036 | 8 | 89 | 30 | 25 | 152 | 5% | 1 | 8 | 8 | 164 | 10 | 191 | 6% |
| royalblue | 561 | 0 | 0 | 122 | 80 | 202 | 36% | 0 | 0 | 0 | 187 | 8 | 195 | 35% |
| saddlebrown | 65 | 0 | 0 | 4 | 0 | 4 | 6% | 0 | 0 | 0 | 18 | 0 | 18 | 28% |
| violet | 36 | 0 | 0 | 12 | 1 | 13 | 36% | 0 | 0 | 0 | 0 | 0 | 0 | 0% |
| blue | 1581 | 4 | 8 | 21 | 11 | 44 | 3% | 7 | 41 | 31 | 0 | 0 | 79 | 5% |
| green | 2387 | 0 | 1 | 207 | 28 | 236 | 10% | 0 | 3 | 0 | 194 | 54 | 251 | 11% |
| steelblue | 64 | 0 | 0 | 6 | 1 | 7 | 11% | 0 | 0 | 2 | 0 | 0 | 2 | 3% |
| paleturquoise | 41 | 0 | 0 | 5 | 0 | 5 | 12% | 0 | 0 | 0 | 0 | 0 | 0 | 0% |
| grey | 266 | 0 | 0 | 0 | 1 | 1 | 0% | 0 | 0 | 0 | 1 | 1 | 2 | 1% |
| Module Sum | 10012 | 42 | 407 | 407 | 146 | 1002 | 10% | 85 | 168 | 172 | 563 | 72 | 1060 | 11% |
| % of Cluster 🡪 | | 78% | 93% | 91% | 90% | 91% | Cluster % 🡪 | 90% | 84% | 83% | 91% | 95% | 88% |  |

Each cell represents the number of transcripts shared between respective module (row) and cluster (column). The “n” column represents the number of transcripts in a given module, and the “n” row represents the number of transcripts in a given cluster. Values in the “Sum” columns are row sums, e.g. the total number of transcripts in each respective module also present in the clusters. The “% of module” columns represent percentage values calculated by dividing the “Sum” columns by the total number of transcripts in a cluster set (1106 for B clusters, and 1198 for P clusters). Values in the “Sum” row are column sums, e.g. the total number of transcripts in each respective cluster that are also present in the modules. The “cluster %” row represent percentage values calculated by dividing the “Sum” row by the total number of transcripts among all modules (10012).
